# Engineering Murine Cross‐Reactivity Into an Affibody to Human Death Receptor 5

**DOI:** 10.1002/bit.70216

**Published:** 2026-05-06

**Authors:** Tse‐Han Kuo, Nagamani Vunnam, Jonathan N. Sachs, Benjamin J. Hackel

**Affiliations:** ^1^ Department of Chemical Engineering and Materials Science University of Minnesota ‐ Twin Cities Minneapolis Minnesota USA; ^2^ Department of Biomedical Engineering University of Minnesota – Twin Cities Minneapolis Minnesota USA

## Abstract

Interspecies cross‐reactive protein therapeutics that target conserved epitopes across species are critical for translational research. The present study showcases the engineering of an affibody molecule, originally discovered for binding to human death receptor 5 (hDR5) with 94 nM affinity, to simultaneously acquire cross‐reactivity to murine DR5 and enhance its binding affinity to human DR5. DR5 plays a pivotal role in metabolic dysfunction‐associated steatohepatitis (MASH) by mediating hepatocyte apoptosis and inflammation. Utilizing a rationally designed library guided by enrichment information and a helix‐walking mutagenesis strategy, combined with alternating binding selections between human and murine DR5, we evolved affibody variants exhibiting significantly improved binding to both receptors. Deep sequencing revealed amino acid preferences in the paratope, and the dominant variant, ABY_DR5‐A_, demonstrated over 1000‐fold and 16‐fold affinity improvements to murine and human DR5, respectively, with equilibrium dissociation constants of 15 and 5.8 nM. ABY_DR5‐A_ exhibited nanomolar IC_50_ values for antagonism of TRAIL‐induced DR5 signaling, measured via caspase 8 activation, in both murine and human cells, albeit with incomplete inhibition. This engineered affibody provides a promising candidate for therapeutic development targeting DR5‐mediated liver disease. Further functional characterization and pharmacokinetic optimization are required to advance these findings toward preclinical evaluation in murine MASH models and, ultimately, clinical applications.

## 1 Introduction

Protein therapeutics that can bind and act on the same molecular target across different species facilitate preclinical evaluation and development. In practice, they are often developed for treating human diseases by interacting with human antigens and binding to the equivalent (orthologous) proteins in mice or primates (Kim et al. 2024a; Lee et al. 2024). This capacity is particularly crucial in the translational study of protein therapeutics because it allows researchers to test the actual drug candidate in preclinical research in animal models of cancers, inflammatory diseases, and autoimmune disorders without converting to a molecular surrogate (Li et al. 2020). This merit largely enhances the predictive power of the preclinical studies. The mechanistic foundation of cross‐species reactivity lies in targeting the conserved binding sites (epitopes) of the orthologous antigens or engineering the protein binders to accommodate the difference in antigen sequence (Lee et al. 2024). In addition to the translational potential, the cross‐reactivity of protein therapeutics also facilitates regulatory approval pathways. Having a cross‐species reactive drug means the same molecule can be examined in two animals (US FDA 2024; Research 2024), which may be required by global regulators, leading to more relevant safety margins.

Successful cases of cross‐reactivity engineering have been shown in several fields, spanning cancer, inflammatory disease, and autoimmune disorder. Moreover, both antibody‐based therapies and alternative protein scaffolds are used in cross‐reactive format. In cancers, utilizing species‐cross‐reactive protein therapeutics represents a chance to test the exact drug in realistic, immune‐competent models. For example, a cross‐reactive anti‐CTLA‐4 antibody (mAb146) was created to bind both human and murine CTLA‐4 (Li et al. 2020). Specifically, in the engineering process, human CTLA‐4 and murine CTLA‐4 extracellular domain (ECD) were alternately employed to immunize rats. The resultant hybridomas were further selected for binding to human, murine, and monkey antigens. The docking modeling reveals overall similar interactions of the mAb to human and murine CTLA‐4. Another case demonstrates a human/murine cross‐reactive anti‐PD‐1 antibody (GNUV201) (Kim et al. 2024a), which was selected for hPD‐1 binding post immunization using human PD‐1 ECD and human PD‐1 expressing cells (Kim et al. 2024a). The molecule exhibits a specific binding to human PD‐1 but not to other immune checkpoint proteins in the same superfamily. Comparable binding was observed to murine PD‐1, showing EC_50_ values of 117 pM and 203 pM to human and murine antigen, respectively. The epitope mapping result displays that GNUV201 targets a well‐conserved FG loop region of hPD‐1 and mPD‐1, supporting the inter‐species cross‐reactivity of the mAb.

Outside the oncology realm, species‐cross‐reactive protein therapeutics have also shown potency in inflammatory diseases. Adalimumab is the first FDA‐approved human mAb discovered via phage display technology (Park et al. 2019; Scheinfeld 2003). The antibody is capable of neutralizing a pro‐inflammatory cytokine, tumor necrosis factor alpha (TNF‐α). Through targeting a conserved epitope on TNF‐α, the binder can bind to and neutralize the antigen from humans, cynomolgus monkeys, dogs, and mice (Ubah et al. 2019; Yu et al. 2017). Though this merit of cross‐reactivity is not from engineering intention but a fortunate outcome, the mAb still renders direct translational insight and is approved worldwide to treat several autoimmune disorders. Human receptors can also be engineered to acquire cross‐species specificity to quench the disease‐associated ligands as decoy: Etanercept is an Fc‐fusion of human TNF receptor 2 ligand binding domain to trap TNF‐α in approved indications in RA and other autoimmune disorders. The structural homology between orthologous TNF‐α molecules provides inhibitory potency in wild‐type mice as well (Venegas‐Pont et al. 2010). Furthermore, alternative protein scaffolds have also exhibited cross‐reactivity. Designed ankyrin repeat proteins (DARPins) were engineered to bind to CD‐4 and, separately, Mac‐1/integrin CD11b/CD18, and the specificities span human/rhesus monkey and human/mouse, respectively (Pugach et al. 2010; Siegel et al. 2021). Fibronectin‐based scaffold, CT‐322 Adnectin, was engineered to recognize both human and mouse VEGFR2 resulting in the ability to test in immunocompetent mouse tumor models (Getmanova et al. 2006). Lastly, affibodies have also been engineered to bind to human and murine transferrin receptor‐1 for brain shuttling purposes using phage‐display with alternate antigen selection (Hjelm et al. 2023). Thus, antibodies and alternative scaffold binders have been engineered for species cross‐reactivity to disease‐associated targets, facilitating preclinical evaluation.

**Figure 1:**
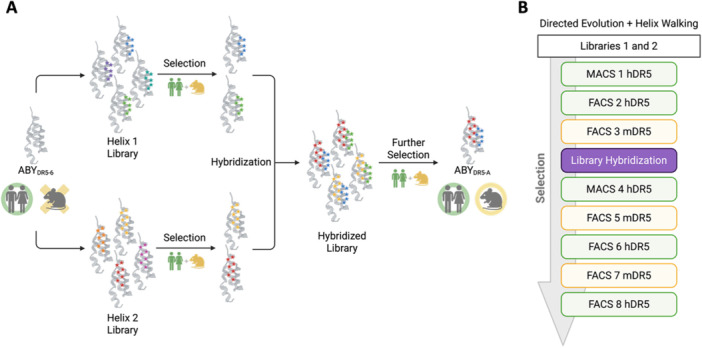
The engineering of human/murine DR5 cross‐reactivity is achieved by combining rational library design, helix‐walking mutagenesis, and directed evolution. (A) The cross‐reactivity engineering began by diversifying a known human DR5 binder, ABY_DR5‐6_, at conventional affibody binding sites within helices 1 and 2. To inform the selection of amino acids at these sites, we surveyed the amino acids that are enriched in previous affibody binders and natural homologous proteins. A helix‐walking mutagenesis approach was adopted, resulting in the creation of two independent libraries. Helix 1 Library possessed diversifications only in the first helix, and Helix 2 Library held diversifications solely in the second helix. These libraries were evolved in parallel through binding selection using human and murine DR5. The evolved helices from the enriched binders from each library were then hybridized. Resultant variants were further sorted for cross‐reactivity and affinity. (B) Schematic illustration of the selection process of human/murine DR5 cross‐reactive binders via directed evolution combined with helix walking mutagenesis. Magnetic‐activated cell sorting (MACS) and fluorescent‐activated cell sorting (FACS) utilizing cell lysate containing human and murine DR5 were employed to isolate the cross‐reactive binders.

In a previous study, our collaborative team discovered an affibody molecule, ABY_DR5‐6_, that can bind to and inhibit human death receptor 5 (DR5) (Vunnam et al. 2020). DR5 is a mediator protein that contributes to hepatocyte cell death and inflammation, contributing to metabolic steatohepatitis (MASH, formerly NASH). MASH is characterized by liver injury (often via lipoapoptosis) and a proinflammatory environment that promotes fibrosis, and DR5 plays a crucial role in these processes (Cazanave et al. 2011; Hirsova et al. 2013; Mori et al. 2004). Overexpression of DR5 has been identified in human samples and animal models (Hirsova et al. 2017). Given the central role of DR5 in hepatocyte apoptosis and the downstream signaling pathways of inflammation and fibrosis, it represents an attractive therapeutic target for MASH. Currently, no drug specifically targeting DR5 in MASH has reached clinical trials, which makes ABY_DR5‐6_ a novel candidate. It binds to hDR5 with a moderate affinity (K_D_ = 94 nM) and inhibits apoptosis via unique non‐competitive, allosteric antagonism. Nevertheless, weak binding to murine DR5 (K_D_ = 15 µM in a preliminary result) hinders its study in preclinical mouse MASH models. In addition, the moderate affinity to hDR5 could potentially be a pitfall in the clinical use (Marei et al. 2022; Auerbach 2016). In the present study, we simultaneously mature hDR5 binding affinity and acquire interspecies cross‐reactivity to murine DR5 (Figure 1A). A tailored library design guided by previous enrichment information in the natural and synthetic homologs was combined with a helix‐walking mutagenesis strategy and rational directed evolution utilizing human and murine antigens (Figure 1B).

## 2 Results

### 2.1 Sequence and Structural Alignments Suggest Moderate Similarity Between Murine and Human DR5

**Figure 2:**
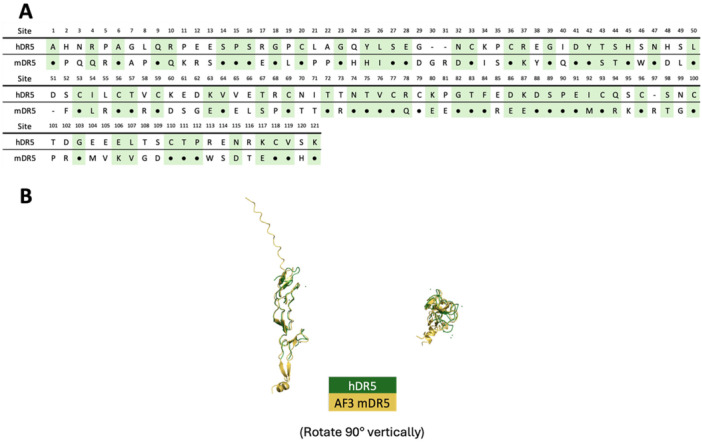
Sequence and structural similarities between human and murine death receptor 5 (DR5) were quantified. (A) The ECD sequence of mDR5 was aligned to the ECD sequence of hDR5 using protein–protein BLAST (blastp). Sites with identical (denoted with •) and homologous amino acids are shown with green overlay. (B) AF3‐predicted ECD structure of mDR5 was structurally aligned to an experimentally‐solved ECD structure of hDR5 (PDB: 1DU3), revealing an RMSD = 0.85 Å and a TM‐score = 0.86.

To assess the feasibility of engineering human/murine cross‐reactive DR5 binding, we evaluated the similarities in sequence and structure between the receptors. Murine DR5 extracellular domain is 38% identical and 57% homologous to the human counterpart (Figure 2A). The protein–protein interaction occurs not just in sequence space but in three‐dimensional space; thus, structural alignment between the receptors was performed as well. Since the murine DR5 ECD structure has not been determined experimentally, we employed AlphaFold 3 (AF3) (Abramson et al. 2024) to predict the structure, which yielded a moderately confident prediction (pTM = 0.71). We aligned the hDR5 ECD (PDB: 1DU3) to AF3‐predicted mDR5 ECD, revealing high structural homology characterized by an RMSD of 0.85 Å and a TM‐score (TM‐align Server 2025) of 0.86 (Figure 2B). We also considered functional homology. Upon binding by the cognate ligand, human or murine TRAIL, both human and murine DR5 can be activated to form higher‐order clusters, which in turn elicit downstream signaling (Wajant 2003; Guicciardi and Gores 2009; Wu et al. 1999; Toffoli et al. 2021). Notably, the human ligand TRAIL can bind to and activate both human and murine receptors (Wu et al. 1999; Toffoli et al. 2021). While this indicates structural and functional homology are possible, the lead molecule ABY_DR5‐6_ binds at a distinct epitope from TRAIL as evidenced by its lack of competition (Vunnam et al. 2020). AF3 prediction of the complexes of ABY_DR5‐6_ with either hDR5 or mDR5 did not yield confident structures (iPTM = 0.25 or 0.15, respectively).

To further assess the potential for receptor cross‐reactivity in this family, we note that hTRAIL is naturally cross‐reactive to not only the murine orthologous receptor, mDR5, but also to the human paralogous receptor, hDR4. Thus, several members of the receptor family—including our targets mDR5 and hDR5—have sufficient homology for cross‐reactivity by a natural ligand, which supports the potential for protein binders to be engineered for cross‐reactivity in this family. hDR4 and hDR5 have 61% identity and 66% homology. Structural alignment (hDR4: 5CIR and hDR5: 1DU3) exhibits an RMSD of 0.81 Å and a TM‐score of 0.83. Collectively, the results suggest that engineering cross‐reactive affibodies for human and murine DR5 is a viable option.

### 2.2 The Affibody Library Is Rationally Designed With Enrichment Information and Helix Walking

We performed directed evolution on the parental hDR5 binder, ABY_DR5‐6_, which has a binding affinity to hDR5 of 94 nM and mDR5 of ~15 µM (Vunnam et al. 2020). In engineering binding and functional proteins, our team and others have shown that rationally designed libraries guided by sequence enrichment and structural information to limit and bias the diversity at certain sites can increase the rate of discovering variants with higher fitness (e.g., binding affinity, catalytic activity, and developability) compared to the traditional random mutagenesis libraries that utilizes NNK codon degeneracy or error‐prone PCR (Woldring et al. 2017; Simons et al. 2020; Chen et al. 2012). Hence, we incorporated multiple inputs for rational design of the degree of diversification at each site and the permitted amino acids. The mutations were focused on the 13 conventional affibody binding sites, specifically sites 9, 10, 11, 13, 14, 17, and 18 in helix 1, and sites 24, 25, 27, 28, 32, and 35 in helix 2. Mutation of non‐interfacial sites, including helix 3 and other sites within helices 1 and 2, also have the potential to aid binding, albeit with less frequent benefit than interfacial sites (Lee and Goodey 2011). Thus, we opted to focus on the aforementioned sites to aid efficiency. Paratope sites that are more exposed to the outer environment have a higher chance of interacting with the target; thus, sites with ≥ 60% solvent accessible surface area (SASA) and a sidechain pointing toward the presumed target binding interface were allowed to encode all 20 amino acids. The diversification at other sites was limited to 4–12 different amino acids to control the overall size of the libraries. To guide diversity in these sites, we incorporated amino acids enriched at each site in binders to a panel of targets by Woldring and team (2017). We then added the amino acids exhibiting the highest frequencies in natural homologs and other published binders. In select cases, we also included an amino acid with complementary physicochemistry to broaden diversity. The library design was finalized upon consideration of these factors in the context of cost‐efficient degenerate codon synthesis (Table 1 and Supporting Information Table S1). Sites 10, 14, 18, 24, 25, 32, and 32 were encoded to all 20 amino acids. Site 9 encoded D as parental, V and L for strong enrichment in first and second generation binders from the sitewise libraries (Woldring et al. 2017), I for frequency in natural homologs, and N and H from codon degeneracy. Site 11 encoded L as parental, N for enrichment in second generation and other published binders, as well as frequency in natural homologs, hydrophobic I as a homolog, and hydrophilic S and T to broaden chemical diversity. Site 13 encoded V as parental; I for enrichment in all prior binder discovery, F and Y for enrichment in second generation binders and frequency in homologs, and L for homology. Site 17 encodes V as parental, hydrophobic I, L, and F and hydrophilic D, N, H, Y, A, T, and S. Site 27 encodes T as parental, I and V for enrichment in second generation and published binders, and A from codon degeneracy. Site 28 encodes L as parental, F, I, and N for enrichment in binders, mid‐hydrophilic S and T for physicochemical diversity (as site 28 is in the middle of the diversified region), and H and Y from codon degeneracy. To achieve reasonable sampling of the designed sequence space, we employed a helix‐walking strategy, that mimics CDR walking, to conduct the mutagenesis on ABY_DR5‐6_. This approach has been shown to (1) preserve the original functions of the parental proteins in the engineering process, (2) allow for the exploration of a deeper and more focused space in each site in the epitopes, and (3) combine affinity gain in each walked region and incorporate epistasis between regions (Yang et al. 1995; Stern et al. 2019; Colley et al. 2018). Consequently, we grouped the aforementioned design options in each site based on their locations on the affibody scaffold and created an independent library for each of the two paratope helices with a theoretical diversity of 5.9 × 10^7^ for the Helix 1 Library and 3.8 × 10^7^ for the Helix 2 Library (Table 1). The library DNA was transformed into EBY100 yeast for yeast surface display resulting in 6.4 × 10^7^ and 6.6 × 10^7^ transformants for Helix 1 and Helix 2 Libraries, revealing 66% and 79% probabilities to sample each designed variant in each library, respectively. Deep sequencing revealed 99% accuracy to diversity design in both Helix 1 Library 1 (Supporting Information Figure S1A) and Helix 2 Library (Supporting Information Figure S1B), as well as 99% accuracy of framework conservation in each (Supporting Information Figure S1C,D). Lastly, the amino acid occurrence frequency in the diversified sites was comparable to the theoretical library design (Supporting Information Figure S1E,F).

**Table 1:** A helix‐walking mutagenesis technique and a rational library design were employed to discover human/murine DR5 cross‐reactive binders.

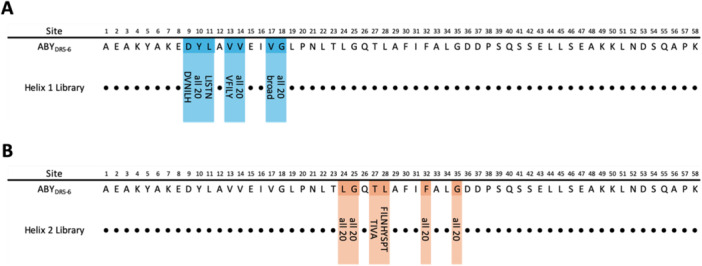

*Note:* In diversifying the 13 conventional paratope sites within the affibody, sites with greater than 70% solvent‐accessible surface were allowed to mutate to all 20 amino acids. Other sites were mutated to 4–12 amino acids informed by the amino acid preferences in affibody homologs and previously evolved affibody binders. To keep the parental human DR5 binding at a baseline level and explore a greater depth in the mutational fitness space, we employed a helix‐walking mutagenesis strategy that mimicked CDR‐walking. Specifically, diversity in helices 1 and 2 was divided into two independent libraries. (A) Design of Helix 1 Library. (B) Design of Helix 2 Library. “all 20” indicated that the site was allowed to be mutated to all 20 canonical amino acids. “broad” indicated that the site can be mutated to A, D, F, H, I, L, N, P, S, T, V, and Y.

### 2.3 Initial Sorting Enriches Cross‐Reactive Binders to Human and Murine DR5

As targets for directed evolution, we identified sources of full‐length, membrane‐integrated cellular human and murine DR5 from mammalian cell lines. HEK293 cells stably transfected with an expression vector for hDR5 with the cytoplasmic domain replaced by green fluorescent protein (GFP) (HEK293‐DR5) (Vunnam et al. 2020) were quantified to express 1.2 × 10^6^ human DR5 per cell using anti‐hDR5 mAb and a bead quantification kit (Supporting Information Table S4). Mouse fibroblast L929 cells are models for several murine DR5 studies with constitutive murine DR5 expression (Dufour et al. 2017; Hilliard et al. 2001; Thon et al. 2006). Absolute quantification of murine DR5 expression on the L929 cells could not be conducted due to the lack of a kit‐compatible mAb against murine DR5 at the time of the experiment. Nevertheless, the expression of the receptor was verified via flow cytometry (Supporting Information Figure S2). For the negative target to deplete non‐specific binders in magnetic‐activated cell sorting (MACS), human DR4 was used due to its homologous role to DR5. DR4 and DR5 proteins with cytoplasmic GFP fusions were prepared as detergent‐solubilized lysates of transiently‐ and stably transfected HEK293 cells (Supporting Information Figure S3A,D), respectively. Receptors were conjugated to magnetic beads with immobilized GFP‐binding nanobodies (Supporting Information Figure S3B,C,E,F).

**Figure 3:**
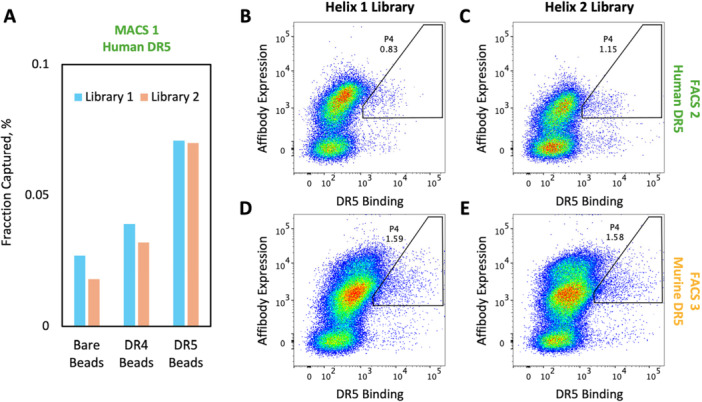
Helix 1 and Helix 2 libraries were evolved in parallel for human and murine DR5 cross‐reactive binding. (A) Nonspecific binders in the initial Helix 1 and Helix 2 libraries were depleted by bare GFP‐trapping magnetic beads and DR4‐coated magnetic beads. Human DR5 binders in the populations were then isolated by DR5‐coated magnetic beads. (B and C) Yeast‐displayed libraries were incubated with 250 nM biotinylated human DR5 and 17 nM anti‐C‐myc mAb, washed, and labeled with 17 nM streptavidin Alexa Fluor 488 conjugate and 17 nM goat anti‐mouse pAb Alexa Fluor 647 conjugate. The top 0.8%–1.2% of variants exhibiting high affibody expression and strong binding to human DR5 were collected. (D and E) After expansion from FACS2, yeast‐displayed libraries were incubated with onefold of biotinylated murine DR5 and 17 nM anti‐C‐myc mAb, washed, and labeled with 17 nM streptavidin Alexa Fluor 488 conjugate and 17 nM goat anti‐mouse pAb Alexa Fluor 647 conjugate. The top 1.6% of the variants that were high affibody expressers and potent mouse DR5 binders were binned and isolated.

The diverse affibody libraries were displayed on the yeast surface, and non‐specific binders were depleted using beads coated with anti‐GFP nanobodies and DR4. From the remaining yeast, those displaying affibodies binding hDR5‐conjugated beads were isolated (Figures 1B and 3A). Approximately 0.07% of yeast were collected from each Helix 1 and Helix 2 Libraries, which is reasonable given the large theoretical diversity of Libraries 1 and 2, and the significant deviation, relative to the parental ABY_DR5‐6_ sequence, of amino acids in those fully diversified sites. To further apply selection with higher stringency and better tunability, we employed fluorescent activated cell sorting (FACS). To simultaneously mature the affinity to hDR5 and engineer mDR5 cross‐reactivity, we alternated the target (hDR5 or mDR5) in different rounds of FACS (Figure 1B). The approach has successfully developed cross‐reactivity in antibodies and small protein scaffolds (Getmanova et al. 2006; Kwok et al. 2014; Hartmann et al. 2023; Kim et al. 2024b). The hDR5 binders from MACS were expanded and subjected to FACS for binding with 250 nM hDR5. Approximately 1% of the populations in each library that showed the strongest affibody expression‐normalized hDR5 binding were collected (Figure 3B,C). The binders were expanded and further sorted for mDR5 binding using the detergent‐solubilized lysate of 10 million L929 cells in 0.1 mL (onefold dilution; see Methods for concentration details). While numerous variants did not exhibit appreciable binding to mDR5, there was a significant population with moderate cross‐reactivity; affibody variants that possessed the strongest expression‐normalized mDR5 binding (~1.6% of each population) were isolated (Figure 3D,E).

### 2.4 Hybridization of Enriched Helices 1 and 2 Enable Strong Cross‐Reactive DR5 Binding

**Figure 4:**
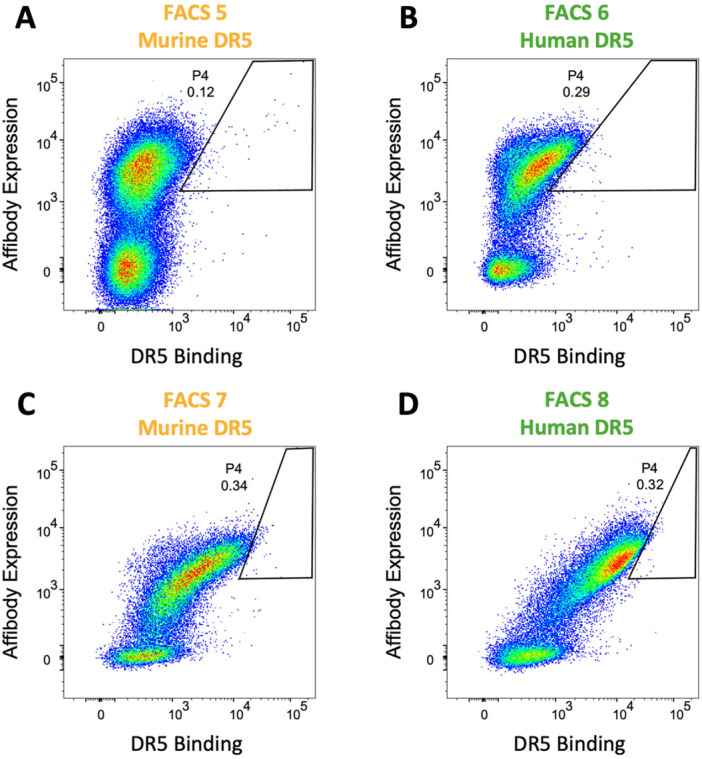
Human and murine DR5 cross‐reactive binders were enriched in the hybridized library through directed evolution. The resultant variants upon hybridizing diversified helix 1 sequences and the diversified helix 2 sequences were subjected to more stringent selections. (A) The library was incubated with 0.2‐fold of biotinylated murine DR5 from cell lysate, washed, and labeled with 17 nM streptavidin Alexa Fluor 488 conjugate and 17 nM goat anti‐mouse pAb Alexa Fluor 647 conjugate. The top 0.12% of the affibody expressers and murine DR5 binders were isolated and expanded. (B) The library was incubated with 12.5 nM biotinylated human DR5 from cell lysate, washed, and labeled with 17 nM streptavidin Alexa Fluor 488 conjugate and 17 nM goat anti‐mouse pAb Alexa Fluor 647 conjugate. The top 0.29% of affibody expressers and human DR5 binders were collected and expanded. (C) The library was incubated with 0.01‐fold of biotinylated murine DR5 from cell lysate, washed, and labeled with 17 nM streptavidin Alexa Fluor 488 conjugate and 17 nM goat anti‐mouse pAb Alexa Fluor 647 conjugate. The top 0.34% of the affibody expressers and murine DR5 binders were isolated and expanded. (D) The library was incubated with 2.5 nM biotinylated human DR5 from cell lysate, washed, and labeled with 17 nM streptavidin Alexa Fluor 488 conjugate and 17 nM goat anti‐mouse pAb Alexa Fluor 647 conjugate. The top 0.32% of the affibody expressers and human DR5 binders were collected and subjected to Illumina deep sequencing.

To explore additive and even synergistic effects, the effective helix 1 sequences enriched from the Helix 1 Library and the effective helix 2 sequences enriched from the Helix 2 Library were hybridized into merged affibody genes via PCR. Yeast transformation resulted in 2.0 × 10^8^ variants, which provides greater than 99% probability of occurrence for each of the 3.2 × 10^6^ potential variants. To also ensure the existence of the improved variants enriched from each parental library, we also included the evolved but not hybridized populations after FACS3. We enriched binders via monovalent MACS using hDR5 as the target with 3.2 × 10^6^ yeasts collected. The binding population was isolated and subjected to four rounds of FACS with alternating mDR5 (at fivefold and 100‐fold dilutions in sorts 5 and 7, Figure 4A,C) and hDR5 (at 12.5 and 2.5 nM in sorts 6 and 8, Figure 4B,D). Despite increasing stringency, the portion of yeasts that showed strong binding to the antigens steadily increased, consistent with evolution of human/murine cross‐reactive binding.

**Figure 5:**
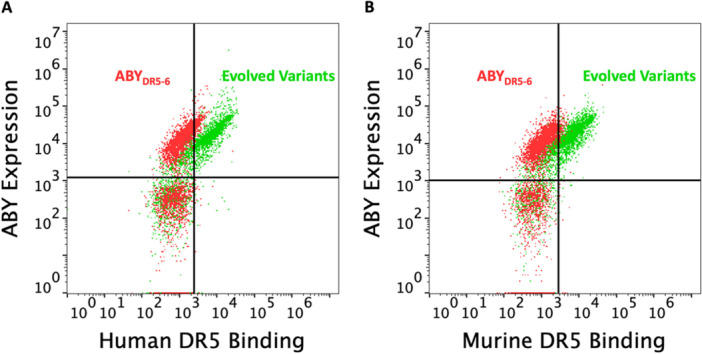
The evolved cross‐reactive variants exhibit improved binding to human and murine DR5 compared to the parental affibody. The evolved variants were expanded and induced after the directed evolution for human/murine DR5 cross‐reactive binding. The yeast‐displayed parental molecule, ABY_DR5‐6_, or the evolved variants were incubated with 10 nM biotinylated human DR5 or 0.04‐fold biotinylated murine DR5 from cell lysate. The yeasts were washed and labeled with 17 nM streptavidin Alexa Fluor 488 conjugate and 17 nM goat anti‐mouse pAb Alexa Fluor 647 conjugate. The evolved variants (green) exhibited matured human (A) and murine (B) DR5 binding compared to the parental molecule (red).

To assess maturation of affinity and human/murine cross‐reactivity, the enriched population was compared to the parental variant, ABY_DR5‐6_, which has a K_D_ of 94 nM to hDR5 and ~15 µM for mDR5 (Vunnam et al. 2020). The evolved library exhibited significantly enhanced binding to both 10 nM hDR5 (Figure 5A) and a 25‐fold dilution of mDR5 (Figure 5B). These findings demonstrate that through a rationally designed library guided by enrichment information, which then incorporates helix walking and directed evolution that alters the target between species, it is possible to mature affinity to hDR5 while simultaneously achieving cross‐reactive binding to mDR5.

### 2.5 The Evolved Hybridized Population Demonstrates Sitewise Amino Acid Preferences

**Figure 6:**
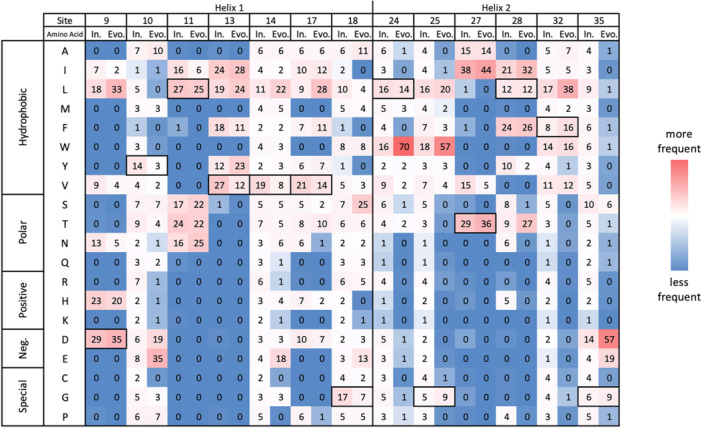
The diversified sites in affibody molecules reveal amino acid preferences in the evolved human/murine DR5 cross‐reactive variants. The amino acid frequencies at each diversified site within the affibody variants enriched for human/murine cross‐reactivity were calculated; amino acids similar in physiochemical properties were clustered together. The amino acids of the parental residues in each mutated site were indicated with thick outer boxes.

To scrutinize the beneficial mutations in maturing the original hDR5 binding and acquiring the novel binding to mDR5, we deep sequenced the evolved variants. Mutational enrichment information was deciphered via sequencing analysis. Of the 76,397 reads from the evolved population post‐FACS8, 22,562 unique amino acid sequences were discovered. Clear preferences can be observed in the 13 diversified sites in the paratope of helices 1 and 2 (Figure 6A). At site 9, parental D remains prevalent as does hydrophilic H; in addition, hydrophobic L is highly enriched (as previously observed in other binders) from 18% to 33% (initial library to cross‐reactive variants). In site 10, parental Y is strongly depleted, and the negatively charged E and D are enriched from 14% to 54%. Site 11 exhibits no strong preferences, suggestive of a lack of target engagement. Sites 13, 14, and 17 deplete parental V despite maintaining or enriching homologs L and I; sites 13 and 14 also enrich a distinct residue (Y13 from 12% to 24% and E14 from 4% to 19%). Site 18 exhibits broad tolerance; the small parental G decreases from 17% to 7% whereas the small, but less conformationally flexible, S and A increase from 7% to 25% and 6% to 11%, respectively. In helix 2 (Figure 6B), sites 24 and 25 exhibit maintenance of their parental amino acids and substantial increase of W. At site 27, A, I, and parental T are maintained whereas V is depleted threefold to 5%. At site 28, polar T is enriched from 9% to 27% yet the more polar S and N are depleted; hydrophobics L and I are maintained and enriched, as is F, yet fellow aromatic Y is fourfold depleted. At site 32, all hydrophobics are tolerated with L strongly enriched; all hydrophilics are depleted. At site 35, despite permitting all 20 amino acids, only four exhibit appreciable presence in cross‐reactive variants; most abundantly, the negatively charged D and E are present at 76% (enriched from 18% in the original library). In summary, amino acid preferences emerged after eight rounds of cross‐reactivity and affinity selections.

### 2.6 The Predominant Evolved Variant, ABY_DR5‐A_, Exhibits Improved Human DR5 Binding and Potent Murine DR5 Cross‐Reactive Binding

**Figure 7:**
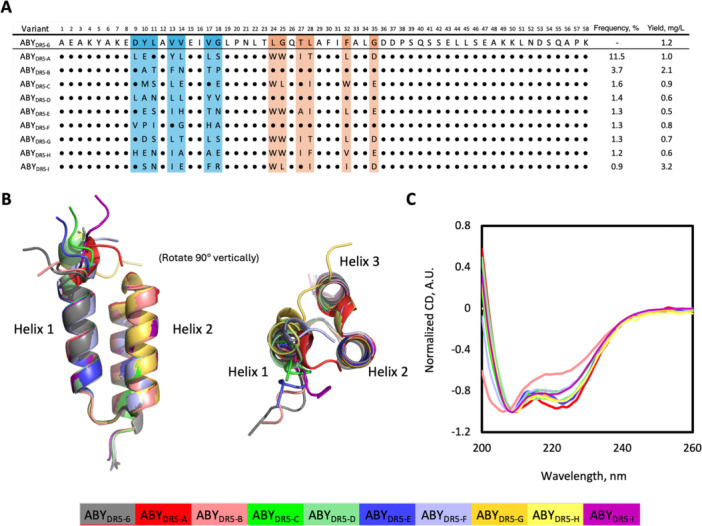
Cross‐reactive affibody molecules are readily expressed and maintain helical structure. (A) The nine most abundant affibodies enriched during cross‐reactive selections are presented in an alignment with the parental molecule, ABY_DR5‐6_. Amino acid sites that are conserved from the parental molecule are denoted with •. Affibodies were produced from pET vectors and purified from the soluble fraction of *E. coli* at 250 mL scale. Yield was quantified using SDS‐PAGE with calibrants. (B) The protein structures of the affibodies were predicted using AlphaFold 3 and aligned with the parental structure, revealing a mean pTM of 0.73 ± 0.03 and a mean RMSD of 0.38 ± 0.10 Å. (C) The secondary structures of the affibodies were assessed using circular dichroism (CD); *n* = 1 for each sample.

We evaluated the nine most frequent variants (ABY_DR5‐A_ to ABY_DR5‐I_) in the evolved cross‐reactive population (Figure 7A and Supporting Information Figure S4A). Two helix 2 motifs are readily apparent: three variants (ABY_DR5‐B_, ABY_DR5‐D_, and ABY_DR5‐F_) conserve the parental helix 2 while the evolved helix 2 in the predominant variant (ABY_DR5‐A_) is matched in another variant (ABY_DR5‐G_) with close homologs in four others. The former result demonstrates the potential importance of helix 2 in interacting with both human and murine DR5 and the merit of helix walking to preserve the parental functions. The latter result highlights the other benefit of helix walking in sampling a broader sequence space to identify more diverse functional sequences.

**Figure 8:**
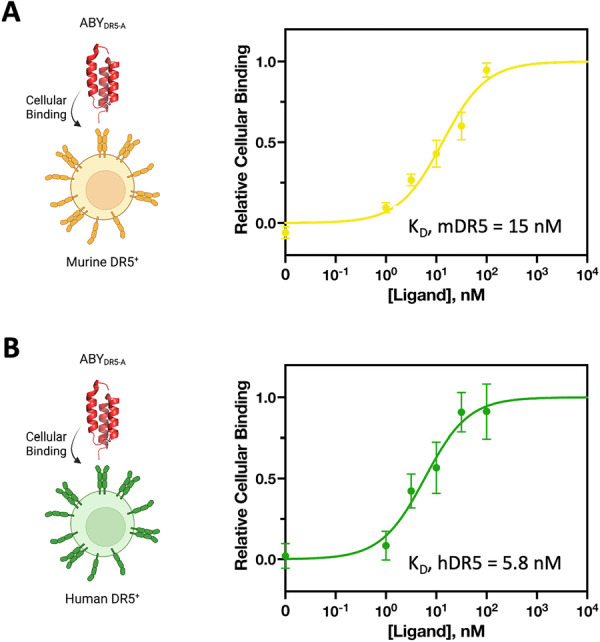
The predominant variant, ABY_DR5‐A_, exhibits improved binding to both murine DR5 and human DR5. ABY_DR5‐A_ was incubated (at 0–100 nM) with murine DR5‐expressing L929 cells (A) or human DR5‐expressing HEK293‐DR5 cells (B). The cells were washed and labeled with 17 nM anti‐His_6_ APC conjugate. Binding was quantified as the median fluorescent signal via flow cytometry. Data are presented as the mean ± standard deviation of four replicates. The equilibrium dissociation constant (K_D_) was determined via a global fitting on all replicates with a 1:1 binding model by minimizing the sum of squared residuals. For murine DR5, ABY_DR5‐A_ bound with a K_D_ of 15 nM (CI: 8–27 nM, *n* = 4). For human DR5, the K_D_ was 5.8 nM (CI, 4–9 nM, *n* = 4).

The nine affibodies were produced in the soluble fraction of *E. coli* bacteria at 0.5–3 mg/L yields without expression optimization (Figure 7A), comparable to the parental molecule, ABY_DR5‐6_. Structures of the affibodies were predicted with AF3 (Abramson et al. 2024) with good confidence (mean pTM = 0.73 ± 0.03). When aligned with the parental (Figure 7B), the result demonstrates overall similar affibody structures (RMSD = 0.38 ± 0.10 Å) with moderate variance in the first few residues (A1 to A6), distal to the evolved paratope. The structures were further probed experimentally via circular dichroism, and all molecules display spectra typical of ⍺‐helices with local minima around 208 and 222 nm, albeit with a less pronounced 222 nm minimum for ABY_DR5‐B_ (Figure 7C). ABY_DR5‐A_ was selected for additional characterization due to its highest prevalence after binder selections, solid yield (Figure 7A and Supporting Information Figure S4A), and maintained secondary structure (Figure 7C). Binding affinities to the two receptor species were quantified via titration on mammalian cells with flow cytometric detection. ABY_DR5‐A_ demonstrates a 15 nM dissociation constant (K_D_) for mDR5 (68% CI: 8–27 nM, Figure 8A), which is an ~1000‐fold improvement from the parental K_D_ of 15 µM. Excitingly, the variant also matures its binding to the original antigen, hDR5, with a K_D_ of 5.8 nM (CI: 4–9 nM, Figure 8B), a 16‐fold enhancement from the parental K_D_ of 94 nM.

A tailored library design combined with helix walking and directed evolution selections alternating between human and murine antigen enabled development of an affibody molecule with human/murine cross‐reactive DR5 binding at low‐nM affinities.

### 2.7 The Predominant Evolved Variant, ABY_DR5‐A_, Exhibits Partial Antagonism of Caspase 8 Activity in Human and Murine Cell Lines

**Figure 9:**
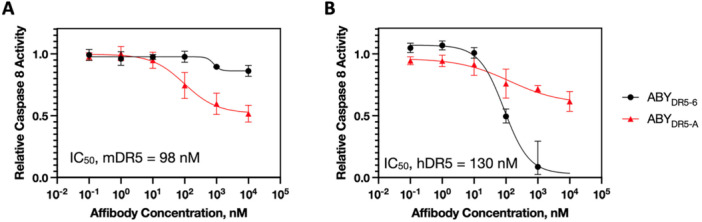
The evolved variant, ABY_DR5‐A_, exhibits cross‐species human/murine inhibition of TRAIL‐induced caspase 8 activity. Murine DR5‐expressing L929 cells (A) and human DR5‐expressing Jurkat cells (B) were incubated for 2 h with 100 pM–10 µM affibody (the most enriched molecule, ABY_DR5‐A_, as well as the parental ABY_DR5‐6_) prior to activation via the cognate ligands, murine TRAIL or human TRAIL, respectively, at 0.1 µg/mL for 16 h. The cells were then assessed for caspase 8 activity via the Caspase Glo‐8 assay. A global fitting on all three replicates with a nonlinear regression of a dose‐response inhibition model was performed to obtain the IC_50_ and maximal inhibition. Data are presented as the mean ± SD. ABY_DR5‐A_ yielded an IC_50_ = 98 nM (CI: 59–160 nM) for murine DR5 (A) and IC_50_ = 130 nM (CI: 34–500 nM) for human DR5 (B).

The cross‐species binding affibody, ABY_DR5‐A_, was then comparatively evaluated with the parental ABY_DR5‐6_ for antagonism of TRAIL‐induced DR5 signaling in murine and human cell lines using caspase 8 activity as the functional output. As expected from its weak murine binding (K_D_ ~ 15 µM), the parental ABY_DR5‐6_ exhibited minimal inhibition (14% maximal suppression) of caspase 8 activation in murine DR5‐expressing L929 cells (Figure 9A). Conversely, ABY_DR5‐A_, engineered for murine/human cross‐reactive binding, demonstrated nanomolar potency (IC_50_ = 98 nM; 68% CI: 59–160 nM) and 48% maximal inhibition on L929 cells. Similar performance was observed for ABY_DR5‐A_ on human Jurkat cells, with IC_50_ = 130 nM (68% CI: 34–500 nM) and 40% maximal inhibition (Figure 9B). The parental ABY_DR5‐A_ maintained its ~100% inhibition on human cells with IC_50_ = 88 nM (68% CI: 73–110 nM). Thus, engineering of murine/human cross‐reactive binding enabled murine/human cross‐reactive antagonism of TRAIL‐induced DR5 signaling—as evaluated via caspase 8 activity—albeit with incomplete maximal inhibition. Future studies will evaluate the mechanistic basis of the modified inhibitory potential, as well as whether the engineered variant maintains the non‐competitive allosteric mode of the parental ABY_DR5‐6_.

## 3 Discussion

Metabolic steatohepatitis (MASH) is a more advanced stage of metabolic dysfunction–associated steatotic liver disease (MASLD) (Hashimoto et al. 2013; Michelotti et al. 2013; Chang et al. 2023). Given the progressive nature of the disease, intervening in the stage of MASH is of great promise to prevent the onset of fibrosis, cirrhosis, and ultimately liver cancers. Nevertheless, only one therapeutic has been approved by the FDA to treat MASH (US FDA 2025); thus, finding novel molecular targets and therapeutics is critical. Our collaborative team has previously discovered a small protein scaffold, ABY_DR5‐6_, that can target and inhibit one of the disease‐associated antigens, DR5, with a moderate K_D_ of 94 nM (Vunnam et al. 2020). Yet, to translate into a more clinically relevant setting, we are concerned that the affinity could be a potential pitfall. Additionally, the molecule binds to mDR5 with a weak affinity (15 µM K_D_), which further precludes its preclinical study in animal models. Aiming to resolve these hurdles, we employed a directed evolution on the parental affibody, ABY_DR5‐6_, for human/murine DR5 cross‐reactive binding that was further implemented with enrichment information‐guided tailored library design and helix walking mutagenesis. The resultant lead molecule, ABY_DR5‐A_, possesses improved affinities to human and murine DR5 with 5.8 nM and 15 nM K_D_, respectively, which is a 16‐ and 1000‐fold enhancement from the parental molecule. We hypothesize that murine affinity enhancement was substantially greater than the human affinity enhancement because of the weaker parental affinity coupled with comparable selection stringencies. Beyond affinity improvement, the functional data further support the translational value of ABY_DR5‐A_. While the parental ABY_DR5‐6_ showed minimal antagonism of TRAIL‐induced DR5 signaling in murine cells, consistent with its weak murine binding, ABY_DR5‐A_ achieved partial inhibition of caspase 8 activation in both murine and human cell lines with nanomolar potency. In murine L929 cells, ABY_DR5‐A_ exhibited an IC_50_ of 98 nM and 48% maximal inhibition, whereas in human Jurkat cells it retained activity with an IC_50_ of 130 nM and 40% maximal inhibition. By comparison, the parental ABY_DR5‐6_ remained highly inhibitory in human cells, with an IC_50_ of 88 nM and near‐complete inhibition, but lacked meaningful murine functionality. Together, these results indicate that engineering cross‐reactive binding successfully translated into cross‐species functional antagonism, thereby overcoming a major limitation of the parental molecule for preclinical evaluation.

Future work could evaluate additional leads; while ABY_DR5‐A_ was the most abundant variant (Figure 7A), ABY_DR5‐F_ displays the highest enrichments in the final murine and human DR5 binding selections, with 21‐ and 10‐fold, respectively, (Supporting Information Figure S4B). The parental variant, ABY_DR5‐6_, is antagonistic to hDR5, inhibiting DR5‐induced caspase‐8 activity with IC_50_ of 160 nM (Vunnam et al. 2020); negligible inhibition was detected in mouse cells, explicable by its weak affinity to the murine receptor. The evolved variant, ABY_DR5‐A_, exhibits cross‐species inhibition with nM IC_50_ values for murine and human DR5‐expressing cells, albeit with 40%–48% maximal inhibition. Incomplete inhibition could result from modified structural engagement. As AF3 fails to confidently model the affibody:DR5 complexes, co‐crystallography and/or epitope mapping will be needed to assess structural drivers of performance. Regardless of inhibitory extent, the high‐affinity cross‐reactive ABY_DR5‐A_ is a strong lead binder for targeted therapy such as proteolysis‐targeting chimeras (PROTACs) (Sun et al. 2019; Li and Crews 2022) or lysosome‐targeting chimaeras (LYTACs) (Banik et al. 2020).

The current work engineered murine/human cross‐reactivity motivated by the value of preclinical murine studies. Evaluation and/or engineering of additional species reactivity, for example, cynomolgus macaque for preclinical toxicology, could also be pursued in the future. Pharmacokinetic modulation could be pursued via PEGylation or fusion to an albumin‐binding domain or antibody Fc domain (Binder and Skerra 2025). The location of the C‐terminus on the third helix as distal to the engineered binding paratope supports this as a potentially effective location for conjugation to avoid hindrance to potency, although conjugate/fusion performance would need to be evaluated.

## 4 Conclusion

Interspecies cross‐reactivity propels the advancement of protein therapeutics in the preclinical and clinical stages. To translate a previously discovered affibody molecule to a more clinically relevant setting for treating DR5‐mediated MASH, we matured hDR5 affinity and murine cross‐reactivity. An enrichment information‐guided tailored library was combined with rationally designed direction evolution and helix walking mutagenesis. The evolved variants demonstrated significantly improved binding to human and murine DR5 compared to the parental molecule. Deep sequencing results showcase amino acid preference in the paratopic sites and reveal the predominant variants. The most prevalent variant, ABY_DR5‐A_, exhibited an over 1000‐ and 16‐fold enhancement to the murine and human receptor at 15 and 5.8 nM K_D_. In the functionality assays, ABY_DR5‐A_ successfully conferred cross‐species functional antagonism of TRAIL‐induced DR5 signaling, yielding partial inhibition in both murine L929 cells and human Jurkat cells with nanomolar potency (murine IC_50_ = 98 nM; human IC_50_ = 130 nM), demonstrating that the engineering goal of gaining murine activity while preserving human activity was achieved. While further characterization of structure and function will be insightful, compelling leads for human/murine cross‐reactive DR5 targeting have been engineered.

## 5 Materials and Methods

### 5.1 Bacterial Strains for DNA Amplification and Protein Production

NEB 10‐beta competent *Escherichia coli* (*E. coli*) (NEB C3019H) were used to amplify the plasmid DNA. BL21(DE3) competent *E. coli* (NEB C2527H) was utilized to produce the soluble affibody protein recombinantly. All *E. coli* strains were purchased from New England Biolabs and cultured by following the manufacturer's protocol. NEB 10‐beta and BL21(DE3) *E. coli* were cultured in lysogeny broth (LB) medium (Fisher Scientific BP1426‐2) with selective antibiotics at 37°C.

### 5.2 Yeast Strains for Yeast Surface Display and Binder Selection

*Saccharomyces cerevisiae* (*S. cerevisiae*) EBY100 (ATCC 50‐238‐3727) were ordered from American Type Culture Collection. EBY100 yeasts were cultured and expanded in yeast extract/peptone/dextrose (YPD) medium (10 g/L yeast extract, 20 g/L peptone, and 20 g/L glucose). After transformation with pCT‐80 yeast surface display plasmid (Stern et al. 2016) via electroporation (Woldring et al. 2017; Benatuil et al. 2010), yeasts were cultured in a selective SD‐CAA medium (16.8 g/L sodium citrate dihydrate, 3.9 g/L citric acid, 20 g/L glucose, 6.7 g/L yeast nitrogen base, and 5.0 g/L casamino acids) at 30°C until optical density at 600 nm (OD_600_) reaches 5.0. Grown yeast was transferred and induced in selective SG‐CAA medium (10.2 g/L sodium phosphate dibasic heptahydrate, 8.6 g/L sodium dihydrogen phosphate monohydrate, 19 g/L galactose, 1 g/L glucose, 6.7 g/L yeast nitrogen base, and 5.0 g/L casamino acids) at 18°C for 20 h to translate and display the affibody protein scaffolds.

### 5.3 Mammalian Cell Lines With DR5 and DR4 Expression

For human DR5 expression and targeting, we used stably transfected HEK293‐DR5 cells (Vunnam et al. 2020). For murine DR5, we chose L929 mouse fibroblast cells (American Type Culture Collection CCL‐1). For negative targets in MACS, we transiently transfected HEK293 cells with human DR4 plasmid: HEK293 cells were grown to 50% confluency and mixed with 23.2 µg DR4‐GFP mammalian cell expression vector DNA (Vunnam et al. 2020) in 1149 µL Opti‐MEM (Gibco 51985091) with 69.6 µL FuGENE 6 Transfection Reagent (Promega E2691). All HEK293 cells were cultured in a completed growth medium with DMEM (Gibco 11965092) supplemented with 10% fetal bovine serum (Gibco A5256701) and 1% penicillin–streptomycin 10,000 U/mL (Gibco 15140122). HEK293‐DR5 cells were additionally cultured under antibiotic selection with 500 µg/mL geneticin (Gibco 1011027). L929 cells were cultured in a completed growth medium with EMEM (ATCC 20‐2003) supplemented with 10% horse serum (Gibco 16050122) and 1% penicillin–streptomycin 10,000 U/mL (Gibco 15140122). All cells were grown to at least 85% confluency prior to trypsinization with TrypLE Express (Gibco 12604039) dissociation reagent for the subjected characterizations. To prepare human and murine DR5 as targeted antigens for FACS, we biotinylated the surface of HEK293‐DR5 and L929 cells with 0.5 mg/mL Sulfo‐NHS‐LC‐Biotin (Thermo Scientific 21335) in phosphate‐buffered saline (PBS) for 0.5 h at room temperature. Biotinylation was stopped by quenching excess biotin molecules with 0.1% albumin in PBS (PBSA) at 4°C. The biotinylated cells were then lysed with native lysis buffer (Abcam ab156035) supplemented with protease inhibitor (Takara Bio 635673) for 20 min with rotation at 4°C. Lysed cells were centrifuged with 15,000 g for 20 min at 4°C to isolate the DR5‐containing cell lysate.

### 5.4 Quantification of DR5 Surface Expression on Mammalian Cells

Trypsinized and harvested HEK293‐DR5 cells were washed 3 times with PBS at room temperature. Approximately 0.2 million cells were labeled with 17 nM anti‐hDR5 mAb (Invitrogen 14990882) in PBSA at 4°C for 1 h with rotation. The primarily labeled cells were then washed with PBSA at 4°C and labeled with 17 nM goat‐anti‐mouse IgG pAb Alexa Fluor 647 conjugate (Invitrogen A21235) in PBSA at 4°C for 0.5 h with rotation. A sample without primary labeling served as a negative control to assess non‐specific secondary antibody binding. The median fluorescent intensity of the labeled cells was measured via flow cytometry. On the same day, four populations of quantification beads with distinct known antibody binding capacities (Bangs Labs 815 A) were labeled with the same labeling protocol. The median fluorescent intensities of the four populations of the beads were recorded via flow cytometry to construct a calibration curve. DR5 expression was interpolated from the curve using background‐subtracted fluorescence intensity of the labeled cells. As for the expression level of murine DR5 on the L929 cells, we could not identify an appropriate monoclonal anti‐mDR5 antibody to quantify the exact copy number of the target. Nevertheless, the expression of murine DR5 was still verified by labeling 0.2 million L929 cells with 17 nM biotinylated anti‐mDR5 pAb (Invitrogen 13588382) in PBSA at 4°C for 1 h with rotation. The primarily labeled cells were then washed with PBSA at 4°C and labeled with 17 nM streptavidin Alexa Fluor 647 conjugate (Invitrogen S32357) for 0.5 h at 4°C with rotation. A sample without primary labeling served as a negative control to assess non‐specific streptavidin binding. The fluorescence intensity of the labeled cells was measured via flow cytometry.

### 5.5 Design and Construction of Yeast‐Displayed Affibody Library

For directed evolution of the human DR5 binder, ABY_DR5‐6_, discovered by Vunnam and team (2020), we rationally diversified 13 conventional sites in the affibody binding paratope. To maintain a baseline level of parental binding to human DR5 and explore a greater depth of sequence space, we employed a CDR‐walking‐mimicking (Yang et al. 1995) mutagenesis technique: helix walking (Stern et al. 2019). We separated the design options for helices 1 and 2 of the affibody into two distinct libraries: helix 1 library had mutations exclusively within helix 1, while helix 2 library 2 contained mutations solely in helix 2 (Supporting Information Table S1). To inform our mutations, we analyzed amino acid preferences at these 13 sites across multiple populations, including homologous proteins of affibodies, published affibody binders, and previous binding campaigns conducted by our team (Woldring et al. 2017). For sites with a solvent‐accessible surface area (SASA) greater than 70%, we allowed diversification to all 20 amino acids. For other sites, we limited mutations to 4–12 amino acids based on the proximity of these amino acids in the codon space. Four overlapping oligonucleotides (IDT, Supporting Information Table S2) encoding the designed diversity were assembled via overlap extension PCR. The PCR product was concentrated with ethanol precipitation and homologously recombined with linearized pCT‐80 vectors (Stern et al. 2016) upon transformation (Woldring et al. 2017; Benatuil et al. 2010) into EBY100 yeast.

### 5.6 Discovery of DR5 Binders Via Magnetic‐Activated Cell Sorting (MACS)

To present the positive target, human DR5, and the negative target, human DR4, on the magnetic beads to perform the selection, we first detached and harvested one T75 flask of stably transfected HEK293‐DR5 or transiently transfected HEK293‐DR4 cells. The cells were washed 3 times with PBS and lysed with 100 µL of native lysis buffer supplemented with protease inhibitor for 20 min with rotation at 4°C. Lysed cells were centrifuged with 15,000 g for 20 min at 4°C to isolate the antigen‐containing cell lysate. One hundred microliters cell lysate was incubated with 15 µL of GFP‐trapping magnetic beads (Chromotek gtma‐20) for 1 h with rotation at 4°C. The mixture was then placed on a magnet for 5 min. The liquid portion was removed entirely, and the beads were resuspended in 15 µL PBSA to create DR5‐ or DR4‐coated beads. Five microliters of human DR5‐ or human DR4‐coated beads were incubated with 17 nM anti‐hDR5 mAb (Invitrogen 14990882) or anti‐hDR4 mAb (Invitrogen 14664482) in PBSA at 4°C for 2 h while rotating. The primarily labeled bead populations were then washed with PBSA at 4°C and labeled with 17 nM goat‐anti‐mouse IgG pAb Alexa Fluor 647 conjugate in PBSA at 4°C for 0.5 h with rotation. The labeled beads were verified for antigen presentations via flow cytometry (Supporting Information Figure S3).

The initial affibody‐displaying yeast libraries, labeled as Libraries 1 and 2, (at a scale of 20 times the number of transformants to provide > 99% confidence of sampling each variant) were incubated with 10 µL of bare GFP‐trapping beads for 2 h. After incubation, the binders that adhered to the beads were removed using a magnet. The unbound populations were then transferred to new tubes containing 10 µL of human‐DR4 coated beads. Following a 2‐h incubation with rotation at 4°C, the human DR4 binders were removed using a magnet. The unbound yeast populations were subsequently transferred to tubes with 10 µL of human‐DR5 coated beads and incubated for an additional 2 h. The human DR5 binders were then isolated with a magnet and expanded for further selections. Throughout these experiments, Libraries 1 and 2 were processed in parallel without mixing.

### 5.7 Discovery of DR5 Binders Via Fluorescent‐Activated Cell Sorting (FACS)

To further conduct sorting on DR5 binders with greater resolution and tunability, we performed several rounds of FACS, which switched targeted antigens between human and murine DR5. The yeast‐displayed populations enriched from Libraries 1 and 2 for hDR5 binding via MACS were grown and induced (at a scale of 20 times the number of cells collected via MACS). Yeast were incubated with biotinylated human or murine DR5 as detergent‐solubilized cell lysate (see above for preparation) and 17 nM anti‐c‐Myc mAb (BioLegend 626802) for 2 h while rotating at 4°C. The primarily labeled yeast were then washed and labeled with 17 nM streptavidin Alexa Fluor 488 conjugate (Invitrogen S11223) and 17 nM goat anti‐mouse pAb Alexa Fluor 647 conjugate with rotation for 30 min at 4°C. After labeling, yeast were resuspended in PBSA at 4°C and sorted via FACS Aria. Among the c‐Myc+ affibody‐expressing populations, variants with the highest DR5 binding: c‐Myc expression ratio (AlexaFluor488:AlexaFluor647) were isolated and expanded. In the human DR5 FACS, the target concentrations were gradually reduced from 250 to 2.5 nM from FACS 2 to FACS 8 to enrich the potent binders stringently. For murine DR5 FACS, because we could not quantify target expression, lysate was varied from undiluted to 100‐fold diluted from FACS 3 to FACS 7. To provide a sense of scale, if the expression was 100,000 mDR5 per cell, this would correspond to 17–0.17 nM; at 1 million DR5 per cell, concentrations would have been 170–1.7 nM.

### 5.8 Discovery of DR5 Binders Via Helix Walking

Libraries 1 and 2 were sorted in parallel for one round of hDR5 MACS, one round of hDR5 FACS, and one round of mDR5 FACS. The enriched affibody‐encoding plasmids from each library were extracted via zymoprep with zymolyase (G Biosciences 786‐914) per the manufacturer's protocol. The gene fragments encoding helix 1 were amplified from the enriched variants of Helix 1 Library via PCR, and the gene fragments encoding helix 2 were amplified from the enriched variants for Helix 2 Library (primers in Supporting Information Table S3). Hybrid genes were assembled by mixing the enriched helix 1 and helix 2 fragments via homologous recombination with linearized pCT‐helix (Woldring et al. 2017) vector upon transformation into EBY100 yeast. Subsequently, one round of human DR5 monovalent MACS was conducted to isolate the binders from the hybridized library: the yeast‐displayed library was incubated with 100 nM human DR5 prepared from cell lysate for 2 h and mixed with 10 µL of GFP‐trapping beads for 2 h while rotating at 4°C. The mixture was placed on a magnet for 5 min, and the binders were isolated and expanded for the ensuing selections. Finally, the library was sorted with gradually increasing stringency for binding to human and murine DR5 in FACS 5 to 8 (Figure 1B).

### 5.9 Identification of Lead Human and Murine DR5 Cross‐Reactive Binding Variants Via Deep Sequencing

We extracted the DNA from enriched variants at several stages of the discovery campaign using zymolyase. The affibody gene was amplified with inner PCR primers (Supporting Information Table S2) and further tagged with Illumina barcodes and adapters in the 5′ and 3′ ends in outer PCR to submit for deep sequencing. DNA was sequenced via Illumina iSeq. Resultant reads were merged, searched, and filtered with USEARCH.

To assess synthesis quality, the initial unsorted Libraries 1 (13,055 reads) and 2 (23,372 reads) were sequenced. Residue frequencies at the diversified sites were compared to the theoretical library design. The amino acids at all 58 sites were analyzed to calculate the unintended mutation rate. The frequency of each unique variant was determined in the final evolved library (post FACS 8 hDR5), resulting in 22,562 unique amino acid sequences identified across 76,397 reads. To compute enrichment (fold‐change in frequency upon a single round of selection), we quantified the occurrence of all variants after the dual‐helix hybrid FACS sorts (post FACS 5 [76,974 reads], 6 [31,945 reads], and 7 [35,955 reads]).

### 5.10 Production and Purification of the Lead ABY_DR5_ Molecules

After identifying the nine most abundant ABY_DR5_ molecules via deep sequencing, we synthesized their genes flanked by overlapping regions to the pET vector at the 5′ and 3′ ends (Supporting Information Table S3). The genes were inserted into the pET plasmid, which includes a C‐terminal His_6_ tag, via HiFi DNA assembly (NEB E2621S) per the manufacturer's protocol. Completed pET plasmid DNA was transformed into BL21(DE3) *E. coli* following the manufacturer's instructions. The transformed *E. coli* was grown in 250 mL LB medium with 50 µg/mL kanamycin (RPI K22000‐25.0) from OD_600_ 0.1 to 0.75 at 37°C while shaking at 250 rpm. Affibody expression was induced with 1 mM IPTG (Teknova 100219‐244) at 18°C for 17.5 h. Cells were centrifuged at 3200 g for 20 min at 4°C and the pelleted cells were resuspended in 3 mL wash buffer (50 mM sodium phosphate, 300 mM sodium chloride, 10 mM imidazole; pH 7.4) supplemented with protease inhibitors (Thermo Scientific A32965). The *E. coli* suspension mixture was sonicated to break down the cell walls and membranes and centrifuged at 15,000 g for 20 min at 4°C to remove the insoluble fraction. The supernatant was added to a 3 mL bed volume of cobalt resin (Thermo Scientific 89964) and washed with 75 mL of wash buffer. Affibody was fractionally eluted with elution buffer (50 mM sodium phosphate, 300 mM sodium chloride, 150 mM imidazole; pH 7.4). The collected eluents were assessed for purity via SDS‐PAGE. Eluents with pure affibody were de‐salted via dialysis in 2 L PBS for two rounds and concentrated by 3 kDa MWCO protein concentrators (Millipore UFC800308). The dialyzed and concentrated affibodies were cryopreserved via liquid nitrogen snap freezing. To quantify affibody concentration, we analyzed the affibody samples along with lysozyme standards via SDS‐PAGE.

### 5.11 Cellular Affinity Titration

Human DR5‐expressing HEK293‐DR5 cells or murine DR5‐expressing L929 cells were detached and harvested from the T75 flasks and washed 3 times with PBS. Approximately 0.2 million cells were incubated with the affibody at six different concentrations. The incubation lasted 3.5 h at 4°C while rotating, allowing for binding equilibrium between the affibody and DR5 molecules. The primarily labeled cells were then washed once with PBS and labeled with 17 nM of anti‐His_6_ APC conjugate (BioLegend 362695) for 30 min at 4°C with rotation. The cells were washed twice with PBSA at 4°C, resuspended in 100 µL cold PBSA, and median fluorescence intensities were measured by flow cytometry. The equilibrium dissociation constant (K_D_) was determined by global fitting of all four replicates to a 1:1 binding model $\left\{F=F_{\mathrm{background}}+\left(F_{\mathrm{sat}}-F_{\mathrm{background}}\right)\left(\frac{C_{\mathrm{ABY}}}{C_{\mathrm{ABY}}+K_{D}}\right)\right\}$ using nonlinear regression to minimize the sum of squared residuals. This model is supported by the use of affibody at dilute concentrations and by the overall quality of fit to the data. Median binding fluorescence values at each affibody concentration were fit globally to obtain a single shared K_D_ across all four replicates, while allowing replicate‐specific maximal and minimal binding fluorescence values. The fitted data were then normalized with replicate‐specific maximal and minimal binding fluorescence for plotting.

### 5.12 Caspase 8 Assay

Jurkat cells were cultured in RPMI 1640 medium (ATCC, 30–2001). Media was supplemented with 2 mM l‐glutamine (Gibco, 25030149), heat‐inactivated 10% fetal bovine serum (Sigma, F0926), 100 U/mL penicillin, and 100 μg/mL streptomycin. Mouse L929 cells (ATCC and CCL‐1) were cultured in EMEM with Earle's balanced salt solution, non‐essential amino acids, 2 mM l‐glutamine, 1 mM sodium pyruvate, and 1500 mg/L sodium bicarbonate. Media was supplemented with 100 U/mL penicillin, and 100 μg/mL streptomycin and 10% horse serum. Jurkat and L929 cells were seeded in 96‐well plates at 5000 cells/well and incubated for 24 h at 37°C. Cells were treated with soluble affibody (1 pM–10 μM), incubated for 1–2 h, and then treated with hTRAIL (Biolegend, 752906) or mTRAIL (BioTechne, 1121‐TL/CF) (0.1 μg/mL), followed by 16 h of incubation at 37°C. An equal volume of Caspase‐Glo 8 reagent (Promega, G8201) was added to each well, and the luminescence was measured after 30 min using a Cytation 3 Cell Imaging Multi‐Mode Reader luminometer (BioTek). For each affibody variant, caspase‐8 activity was measured in murine or human cells across three biological replicates and normalized within each replicate using the no‐TRAIL control and the 0 nM affibody, TRAIL‐stimulated condition. The normalized data were then globally fit using nonlinear regression in GraphPad Prism and Python SciPy (curve_fit). A four‐parameter dose‐response inhibition model $\left\{{\mathrm{Act}}_{\mathrm{norm}}=S_{\min}+\left(S_{\max}-S_{\min}\right)/\left(1+{\left(\frac{C_{\mathrm{ABY}}}{{\mathrm{IC}}_{50}}\right)}^{n}\right)\;\right\}$ was utilized to determine the IC_50_ and maximal inhibition.

## Author Contributions

**Tse‐Han Kuo:** investigation (lead), analysis (lead), writing (equal). **Nagamani Vunnam:** investigation (supporting), writing (supporting). **Jonathan Sachs:** supervision (supporting), writing (supporting). **Benjamin Hackel:** supervision (lead), analysis (supporting), writing (equal).

## Conflicts of Interest

Nagamani Vunnam, Jonathan N. Sachs, and Benjamin J. Hackel have a patent application related to molecules reported in this study.

## Supporting information

Supporting File

## Data Availability

Source data are available on the Hackel Lab's Github repository: https://github.com/HackelLab-UMN.
