# Supplementary material for: Engineering Murine Cross‐Reactivity Into an Affibody to Human Death Receptor 5
Source: Biotechnol Bioeng. 2026 May 6;123(7):1783–98. doi: 10.1002/bit.70216 (PMC13245200; doi:10.1002/bit.70216)
Supplement: Supplementary file 1 — Supporting File [file BIT-123-1783-s001.pdf]

Supporting Information

Interspecies Specificity Engineering of an Affibody Scaffold to Human and Murine Death Receptor 5

Tse-Han Kuo[1], Nagamani Vunnam[2], Jonathan N. Sachs[2], Benjamin J. Hackel[1,2]

[1]Department of Chemical Engineering and Materials Science

[2]Department of Biomedical Engineering

University of Minnesota – Twin Cities

Minneapolis, MN, United States 55455

* Correspondence:

421 Washington Avenue SE

Minneapolis, MN 55455

hackel@umn.edu

Table S1. Table 1 with the addition of encoding DNA: A helix-walking mutagenesis technique and a rational library design were employed to discover human/murine DR5 cross-reactive binders. In diversifying the 13 conventional paratope sites within the affibody, sites with greater than 70% solvent-accessible surface were allowed to mutate to all 20 amino acids. Other sites were mutated to 4 to 12 amino acids informed by the amino acid preferences in affibody homologs and previously evolved affibody binders. To keep the parental human DR5 binding at a baseline level and explore a greater depth in the mutational fitness space, we employed a helix-walking mutagenesis strategy that mimicked CDR-walking. Specifically, diversity in helices 1 and 2 were divided into two independent libraries. (A) Design of Helix 1 Library. (B) Design of Helix 2 Library. “all 20” indicated that the site was allowed to be mutated to all twenty canonical amino acids. “broad” indicated that the site can be mutated to A, D, F, H, I, L, N, P, S, T, V, and Y.

A

Site	1	2	3	4	5	6	7	8	9	10	11	12	13	14	15
ABY$_{DR5-6}$	A	E	A	K	Y	A	K	E	D	Y	L	A	V	V	E
Design Option	•	•	•	•	•	•	•	•	DVNILH	all 20	LISTN	•	VFILY	all 20	•
Degenerate Codon	GCC	GAA	GCG	AAA	TAC	GCT	AAA	GAA	VWC	NNK	WYA AAC	GCG	NTC TAT	NNK	GAG

Site	16	17	18	19	20	21	22	23	24	25	26	27	28	29	30
ABY$_{DR5-6}$	I	V	G	L	P	N	L	T	L	G	Q	T	L	A	F
Design Option	•	broad	all 20	•	•	•	•	•	•	•	•	•	•	•	•
Degenerate Codon	ATC	NHT	NNK	CTG	CCG	AAC	CTG	ACC	CTG	GGT	CAG	ACC	CTG	GCA	TTC

Site	31	32	33	34	35	36	37	38	39	40	41	42	43	44	45
ABY$_{DR5-6}$	I	F	A	L	G	D	D	P	S	Q	S	S	E	L	L
Design Option	•	•	•	•	•	•	•	•	•	•	•	•	•	•	•
Degenerate Codon	ATC	TTT	GCA	CTG	GGT	GAT	GAC	CCG	TCC	CAG	AGC	TCT	GAA	CTC	CTG

Site	46	47	48	49	50	51	52	53	54	55	56	57	58
ABY$_{DR5-6}$	S	E	A	K	K	L	N	D	S	Q	A	P	K
Design Option	•	•	•	•	•	•	•	•	•	•	•	•	•
Degenerate Codon	TCT	GAG	GCG	AAG	AAA	CTG	AAC	GAT	TCC	CAA	GCA	CCA	AAA

B

Site	1	2	3	4	5	6	7	8	9	10	11	12	13	14	15
ABY$_{DR5-6}$	A	E	A	K	Y	A	K	E	D	Y	L	A	V	V	E
Design Option	•	•	•	•	•	•	•	•	•	•	•	•	•	•	•
Degenerate Codon	GCC	GAA	GCG	AAA	TAC	GCT	AAA	GAA	GAC	TAT	CTG	GCG	GTC	GTC	GAG

Site	16	17	18	19	20	21	22	23	24	25	26	27	28	29	30
ABY$_{DR5-6}$	I	V	G	L	P	N	L	T	L	G	Q	T	L	A	F
Design Option	•	•	•	•	•	•	•	•	all 20	all 20	•	TIVA	FILNHYSPT	•	•
Degenerate Codon	ATC	GTC	GGT	CTG	CCG	AAC	CTG	ACC	NNK	NNK	CAG	RYT	HHC	GCA	TTC

Site	31	32	33	34	35	36	37	38	39	40	41	42	43	44	45
ABY$_{DR5-6}$	I	F	A	L	G	D	D	P	S	Q	S	S	E	L	L
Design Option	•	all 20	•	•	all 20	•	•	•	•	•	•	•	•	•	•
Degenerate Codon	ATC	NNK	GCA	CTG	NNK	GAT	GAC	CCG	TCC	CAG	AGC	TCT	GAA	CTC	CTG

Site	46	47	48	49	50	51	52	53	54	55	56	57	58
ABY$_{DR5-6}$	S	E	A	K	K	L	N	D	S	Q	A	P	K
Design Option	•	•	•	•	•	•	•	•	•	•	•	•	•
Degenerate Codon	TCT	GAG	GCG	AAG	AAA	CTG	AAC	GAT	TCC	CAA	GCA	CCA	AAA

Table S2. DNA oligonucleotides used for library gene construction. Amplify5, h1A, h1B, h1C, h1D, h2P, and Amplify3 were used to construct full-length affibody DNA of the Helix 1 Library. Amplify5, h1P, h2A, and Amplify3 were used to construct full-length affibody DNA of the Helix 2 Library. The oligonucleotides were assembled together with three rounds of PCR and concentrated via ethanol precipitation. Amplify5 and ABY1R served as the primers to extract the diversified helix 1 from the yeast plasmids of the evolved Helix 1 Library. ABY2F and Amplify3 served as the primers to extract the diversified helix 2 from the yeast plasmids of the evolved Helix 2 Library. pCT80-ABY FA2N to pCT80-ABY FA5N and pCT-RA2N to pCT-RA5N were used to extract the affibody cassette from the pCT-80 plasmid via PCR (inner PCR). The PCR products were further tagged and extended with FB primers at the 5' end and RPI primers at the 3' end (outer PCR) before submitting to Illumina deep sequencing.

Description	Sequence
Amplify5	GGTGGTGGTGGTTCTGGTGGTGGTGGTTCTGCTAGCGCCGAAGCGAAATACGCTAAAGAA
h1A	GGTCAGGTTCGGCAGMNNADNGATCTCMNNGANCGCTRWMNNGWBTTCTTTAGCGTATTTCGCTTC
h1B	GGTCAGGTTCGGCAGMNNADNGATCTCMNNATACGCGTTMNNGWBTTCTTTAGCGTATTTCGCTTC
h1C	GGTCAGGTTCGGCAGMNNADNGATCTCMNNATACGCTRWMNNGWBTTCTTTAGCGTATTTCGCTTC
h1D	GGTCAGGTTCGGCAGMNNADNGATCTCMNNGANCGCGTTMNNGWBTTCTTTAGCGTATTTCGCTTC
h1P	GGTCAGGTTCGGCAGACCGACGATCTCGACGACCGCCAGATAGTCTTCTTTAGCGTATTTCGCTTC
h2A	CTGCCGAACCTGACCNNKNNKCAGRYTHHCGCATTCATCNNKGCACTGNNKGATGACCCGTCCCAGAGCTCT
h2P	CTGCCGAACCTGACCCTGGGTCAGACCCTGGCATTCATCTTTGCACTGGGTGATGACCCGTCCCAGAGCTCT
Amplify3	TTGGGAATCGTTCAGTTTCTTCGCCTCAGACAGGAGTTCAGAGCTCTGGGACGGGTCATC
ABY1R	GGTCAGGTTCGGCAG
ABY2F	CTGCCGAACCTGACC
pCT80-ABY FA2N	TTTCCCTACACGACGCTCTTCCGATCTNNGGATCTGCTAGCGCCGAAGCGAAAT
pCT80-ABY FA3N	TTTCCCTACACGACGCTCTTCCGATCTNNNGGATCTGCTAGCGCCGAAGCGAAAT
pCT80-ABY FA4N	TTTCCCTACACGACGCTCTTCCGATCTNNNNGGATCTGCTAGCGCCGAAGCGAAAT
pCT80-ABY FA5N	TTTCCCTACACGACGCTCTTCCGATCTNNNNNGGATCTGCTAGCGCCGAAGCGAAAT
pCT-RA2N	GTTCAGACGTGTGCTCTTCCGATCTNNTCGAGCTATTACAAGTCCTCTTCAGAAATAA
pCT-RA3N	GTTCAGACGTGTGCTCTTCCGATCTNNNTCGAGCTATTACAAGTCCTCTTCAGAAATAA
pCT-RA4N	GTTCAGACGTGTGCTCTTCCGATCTNNNNTCGAGCTATTACAAGTCCTCTTCAGAAATAA
pCT-RA5N	GTTCAGACGTGTGCTCTTCCGATCTNNNNNTCGAGCTATTACAAGTCCTCTTCAGAAATAA

Table S3. DNA oligonucleotides used to produce lead affibody variants. The top 9 ABY_{DR5} variants discovered via the human/murine DR5 cross-specific binding campaign were identified via Illumina deep sequencing. The affibody cassettes were then flanked in the 5' and 3' ends by the overlapping regions with the pET vectors to facilitate the assembly process for recombinant affibody production in E. coli.

Description	Sequence
ABYDR5-A	AAGAAGGAGATATACATATGGCTAGCGCCGAAGCGAAATACGCTAAGGAACTGGAGTTGGCATACCTTGAGATC<br>CTTTCCCTGCCCAATTTAACTTGGTGGCAGATCACGGCATTTATCTTGGCCTTAGACGATGACCCTTCACAATCTAGT<br>GAACTGTTGTCGGAAGCGAAGAAATTAAATGATTCCCAAGCACCAAAAGGATCCCACCATCACCATCATCAC
ABYDR5-B	AAGAAGGAGATATACATATGGCTAGCGCCGAAGCGAAATACGCTAAAGAAGATGCTACTGCGTTCAACGAAATTA<br>CCCCGTTGCCCAATCTGACATTGGGTCAAACCCTTGCGTTCATTTTCGCATTAGGAGACGATCCTAGTCAATCGTCC<br>GAGTTACTTTCCGAGGCAAAGAAACTGAATGATTCCCAAGCACCAAAAGGATCCCACCATCACCATCATCAC
ABYDR5-C	AAGAAGGAGATATACATATGGCTAGCGCCGAAGCGAAATACGCTAAAGAAGATATGTCGGCTTTGGAGGAGATT<br>GTCGAATTACCGAATTTGACATGGCTTCAGACTATTGCCTTTATTTGGGCGTTAGAGGACGATCCGTCTCAGTCTTC<br>AGAACTGTTGAGTGAGGCGAAAAAACTGAACGATTCCCAAGCACCAAAAGGATCCCACCATCACCATCATCAC
ABYDR5-D	AAGAAGGAGATATACATATGGCTAGCGCCGAAGCGAAATACGCTAAGGAGTTGGCGAACGCTTTATTAGAAATTT<br>ACGTTCTGCCAAACCTTACGTTAGGCCAAACACTGGCATTTATTTTTGCGTTGGGGGACGATCCGAGTCAAAGCTC<br>GGAGCTGCTGAGCGAAGCCAAAAAGTTAAACGATTCCCAAGCACCAAAAGGATCCCACCATCACCATCATCAC
ABYDR5-E	AAGAAGGAGATATACATATGGCTAGCGCCGAAGCGAAATACGCTAAAGAAGATGAGAGTGCTATCCATGAAATC<br>ACGAATTTACCAAATTTGACTTGGTGGCAGGCCATCGCCTTTATTCTGGCTCTGGAGGACGACCCTTCTCAGTCTAG<br>CGAGTTACTGAGCGAGGCGAAAAAATTGAATGATTCCCAAGCACCAAAAGGATCCCACCATCACCATCATCAC
ABYDR5-F	AAGAAGGAGATATACATATGGCTAGCGCCGAAGCGAAATACGCTAAGGAGGTCCCCATTGCAGTAGGTGAAATT<br>CATGCCTTGCCTAATCTTACTCTTGGTCAGACACTTGCGTTCATCTTTGCCCTTGGGGATGACCCTTCGCAGTCTTCA<br>GAATTATTATCCGAAGCCAAAAAGCTGAATGATTCCCAAGCACCAAAAGGATCCCACCATCACCATCATCAC
ABYDR5-G	AAGAAGGAGATATACATATGGCTAGCGCCGAAGCGAAATACGCTAAGGAAGATGACTCGGCCTTAACGGAGATC<br>CTGTCATTGCCAAATTTGACGTGGTGGCAAATTACGGCTTTTATTTTGGCGCTGGATGATGACCCCTCACAAAGTTC<br>GGAGCTTCTGTCCGAGGCCAAGAAATTAAACGATTCCCAAGCACCAAAAGGATCCCACCATCACCATCATCAC
ABYDR5-H	AAGAAGGAGATATACATATGGCTAGCGCCGAAGCGAAATACGCTAAAGAACACGAGAATGCTATTGCGGAGATC<br>GCCGAACTGCCTAACCTTACATGGTGGCAGATTTTTGCTTTCATCGTCGCCCTGGAGGATGACCCCTCGCAGTCCTC<br>CGAGTTACTTTCAGAAGCCAAGAAGTTGAATGATTCCCAAGCACCAAAAGGATCCCACCATCACCATCATCAC
ABYDR5-I	AAGAAGGAGATATACATATGGCTAGCGCCGAAGCGAAATACGCTAAGGAAGACAGCAACGCGATTGAAGAAATT<br>TTCCGCTTGCCAAACTTGACCTGGTTGCAGACCATTGCATTCATCATCGCCTTGGACGATGACCCGTCGCAAAGCAG<br>CGAATTGTTGTCGGAAGCAAAAAAGTTGAATGATTCCCAAGCACCAAAAGGATCCCACCATCACCATCATCAC

Table S4. Stably transfected HEK293-DR5 cells express human DR5. Cells were labeled by anti-DR5 mouse mAb, washed, and then labeled with goat anti-mouse pAb Alexa Fluor™ 647 conjugate. A sample without anti-hDR5 mouse mAb served as a negative control to assess non-specific binding and background signal. Median fluorescent intensity was recorded. The adjusted (background subtracted) fluorescent signal was converted to cellular target expression by a calibration line constructed by the quantification bead kit.

Sample	Median Fluorescent Intensity	Adjusted Fluorescent Intensity	DR5 expression per cell
HEK293-DR5 + anti-hDR5 mAb	988849	975743	1.2×10^6
HEK293-DR5 − anti-hDR5 mAb	13106		

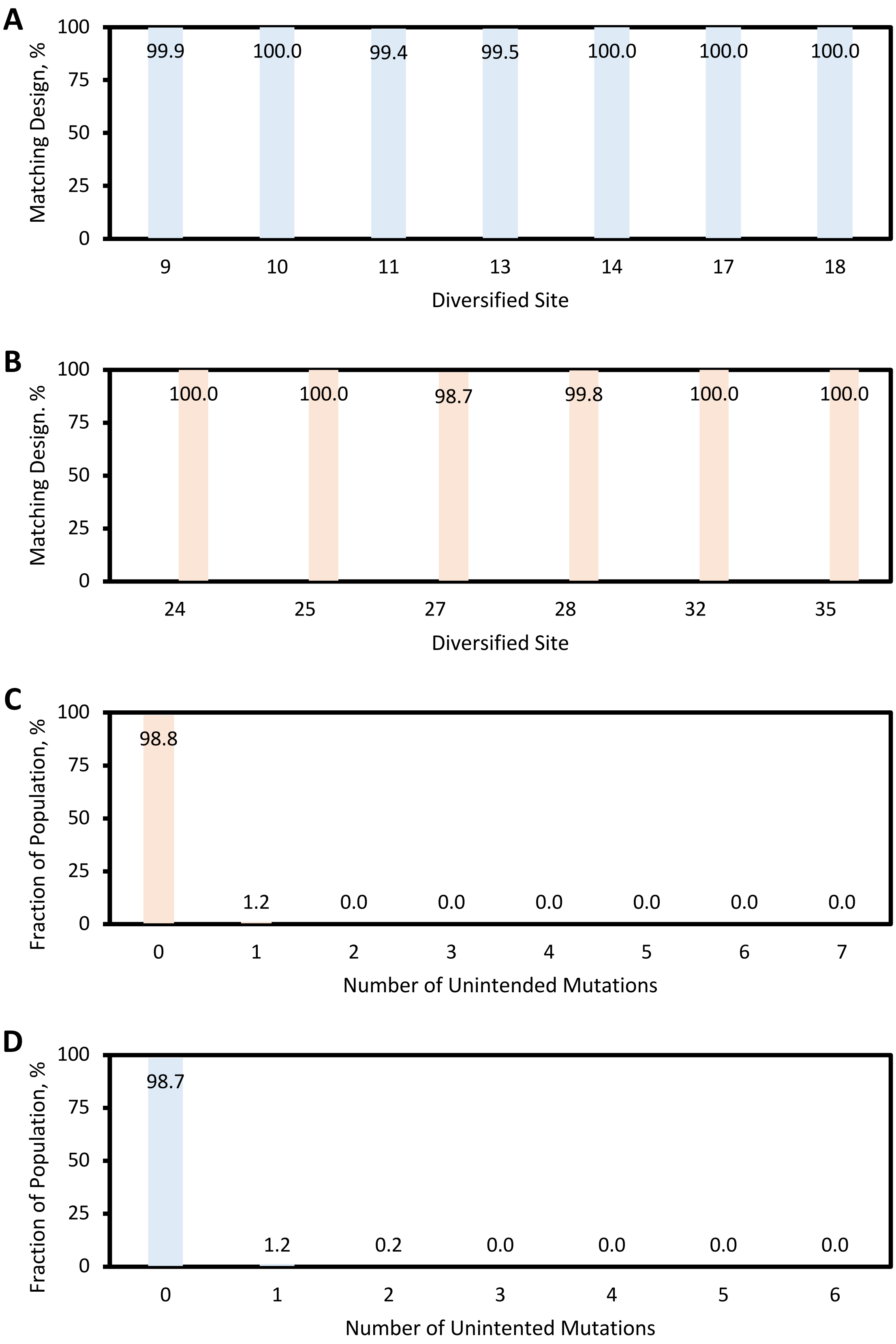
A
Matching Design, %
100
75
50
25
0
99.9
100.0
99.4
99.5
100.0
100.0
100.0
9
10
11
13
14
17
18
Diversified Site
B
Matching Design. %
100.0
100.0
98.7
99.8
100.0
100.0
24
25
27
28
32
35
Diversified Site
C
Fraction of Population, %
98.8
1.2
0.0
0.0
0.0
0.0
0.0
0.0
0
1
2
3
4
5
6
7
Number of Unintended Mutations
D
Fraction of Population, %
98.7
1.2
0.2
0.0
0.0
0.0
0.0
0
1
2
3
4
5
6
Number of Unintented Mutations

E

	Site	9		10		11		13		14		17		18	
	Codon	VWC		NNK		WYA AAC		NTC TAT		NNK		NHT		NNK	
	Amino Acid	theoretical	actual	theoretical	actual	theoretical	actual	theoretical	actual	theoretical	actual	theoretical	actual	theoretical	actual
Hydrophobic	A	0.0	0.0	6.5	6.6	0.0	0.1	0.0	0.1	6.5	5.7	8.3	5.8	6.5	5.9
	I	16.7	7.5	3.2	1.4	20.0	15.6	20.0	24.3	3.2	3.7	8.3	9.8	3.2	1.6
	L	16.7	17.6	9.7	5.1	20.0	26.6	20.0	18.6	9.7	11.5	8.3	9.0	9.7	10.0
	M	0.0	0.0	3.2	3.1	0.0	0.0	0.0	0.0	3.2	4.3	0.0	0.1	3.2	4.5
	F	0.0	0.0	3.2	1.0	0.0	0.5	20.0	17.7	3.2	1.9	8.3	6.7	3.2	1.2
	W	0.0	0.0	3.2	2.8	0.0	0.0	0.0	0.0	3.2	4.4	0.0	0.0	3.2	8.0
	Y	0.0	0.0	3.2	13.8	0.0	0.0	20.0	11.7	3.2	2.2	8.3	5.6	3.2	1.5
	V	16.7	9.2	6.5	4.5	0.0	0.0	20.0	26.5	6.5	19.3	8.3	20.7	6.5	5.3
Polar	S	0.0	0.1	9.7	6.6	20.0	17.3	0.0	0.7	9.7	5.5	8.3	5.1	9.7	6.9
	T	0.0	0.1	6.5	8.8	20.0	23.5	0.0	0.0	6.5	6.6	8.3	7.7	6.5	6.4
	N	16.7	12.8	3.2	2.3	20.0	15.9	0.0	0.0	3.2	2.5	8.3	6.0	3.2	2.1
	Q	0.0	0.0	3.2	3.3	0.0	0.1	0.0	0.0	3.2	2.7	0.0	0.0	3.2	3.3
Positive	R	0.0	0.0	9.7	6.6	0.0	0.0	0.0	0.0	9.7	6.2	0.0	0.1	9.7	6.5
	H	16.7	23.4	3.2	2.5	0.0	0.0	0.0	0.0	3.2	3.1	8.3	6.7	3.2	2.1
	K	0.0	0.0	3.2	2.4	0.0	0.1	0.0	0.0	3.2	2.0	0.0	0.0	3.2	2.1
Negative	D	16.7	29.1	3.2	6.4	0.0	0.0	0.0	0.1	3.2	3.2	8.3	10.0	3.2	2.4
	E	0.0	0.0	3.2	7.6	0.0	0.0	0.0	0.0	3.2	3.6	0.0	0.0	3.2	3.2
Special	C	0.0	0.0	3.2	2.1	0.0	0.0	0.0	0.0	3.2	2.1	0.0	0.0	3.2	3.6
	G	0.0	0.0	6.5	5.5	0.0	0.0	0.0	0.0	6.5	3.4	0.0	0.1	6.5	17.1
	P	0.0	0.1	6.5	6.4	0.0	0.0	0.0	0.1	6.5	4.9	8.3	6.5	6.5	4.8

F

	Site	24		25		27		28		32		35	
	Codon	NNK		NNK		RYT		HHC		NNK		NNK	
	Amino Acid	theoretical	actual	theoretical	actual	theoretical	actual	theoretical	actual	theoretical	actual	theoretical	actual
Hydrophobic	A	6.5	5.5	6.5	4.1	25.0	15.1	0.0	0.2	6.5	4.5	6.5	3.9
	I	3.2	2.9	3.2	3.5	25.0	37.7	11.1	20.7	3.2	4.7	3.2	2.7
	L	9.7	16.0	9.7	16.4	0.0	1.0	11.1	12.2	9.7	16.8	9.7	9.0
	M	3.2	4.6	3.2	4.1	0.0	0.0	0.0	0.1	3.2	4.0	3.2	2.9
	F	3.2	3.6	3.2	7.3	0.0	0.6	11.1	24.2	3.2	8.2	3.2	5.9
	W	3.2	16.4	3.2	17.5	0.0	0.0	0.0	0.0	3.2	14.1	3.2	6.1
	Y	3.2	2.1	3.2	3.0	0.0	0.1	11.1	10.3	3.2	3.7	3.2	2.9
	V	6.5	9.1	6.5	7.5	25.0	15.2	0.0	0.1	6.5	10.6	6.5	5.2
Polar	S	9.7	6.4	9.7	5.4	0.0	0.3	11.1	7.9	9.7	4.8	9.7	9.9
	T	6.5	4.4	6.5	3.3	25.0	28.5	11.1	9.0	6.5	3.1	6.5	4.7
	N	3.2	1.2	3.2	1.5	0.0	0.0	11.1	6.0	3.2	1.4	3.2	2.0
	Q	3.2	1.1	3.2	1.5	0.0	0.0	0.0	0.0	3.2	1.2	3.2	2.0
Positive	R	9.7	4.1	9.7	3.5	0.0	0.1	0.0	0.3	9.7	4.4	9.7	4.8
	H	3.2	1.2	3.2	1.9	0.0	0.2	11.1	4.8	3.2	1.7	3.2	1.9
	K	3.2	0.9	3.2	1.5	0.0	0.0	0.0	0.0	3.2	1.4	3.2	1.2
Negative	D	3.2	4.6	3.2	1.9	0.0	0.4	0.0	0.1	3.2	2.1	3.2	14.1
	E	3.2	3.2	3.2	2.1	0.0	0.0	0.0	0.0	3.2	1.5	3.2	3.9
Special	C	3.2	2.8	3.2	4.5	0.0	0.1	0.0	0.1	3.2	3.5	3.2	3.7
	G	6.5	5.4	6.5	4.5	0.0	0.3	0.0	0.1	6.5	3.9	6.5	6.4
	P	6.5	2.8	6.5	3.0	0.0	0.1	11.1	3.8	6.5	2.7	6.5	5.2

more frequent

less frequent

Figure S1. Transformant sequence identities fall into the theoretical library design. DNA from the initial libraries was extracted from the transformed yeast and submitted for Illumina deep sequencing. **(A, B)** The rate of the amino acid matching the design at each site was calculated by comparing the experimental amino acid identities from the transformants to the theoretical design in 13,055 reads of the initial Helix 1 Library (A) and 23,372 reads of the initial Helix 2 Library (B). **(C, D)** The number of unintended mutations in each sequence read was calculated in all reads from the initial Helix 1 Library (C) and the initial Helix 2 Library (D). **(E, F)** Amino acid frequencies at each site were calculated and compared to the theoretical library design in the initial Helix 1 Library (E) and Helix 2 Library (F).

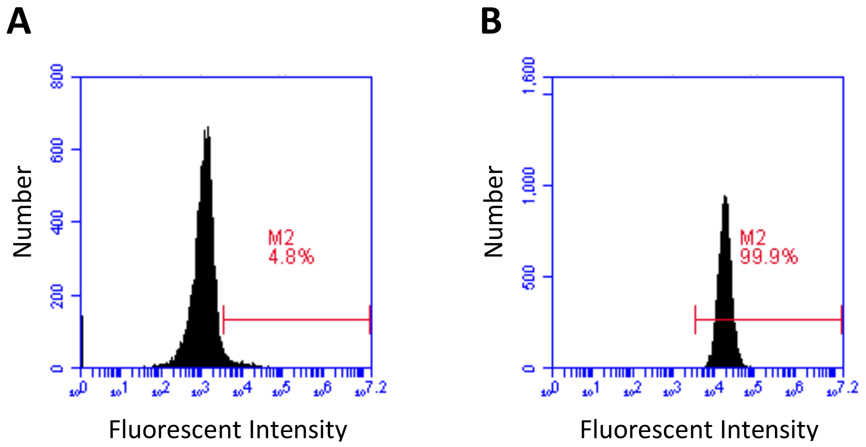

Figure S2. Murine DR5 is expressed on L929 cells. (A) L929 cells were labeled with 17 nM streptavidin Alexa Fluor™ 647 conjugate. **(B)** L929 cells were incubated with 17 nM biotinylated anti-mDR5 pAb and labeled with 17 nM streptavidin Alexa Fluor™ 647 conjugate.

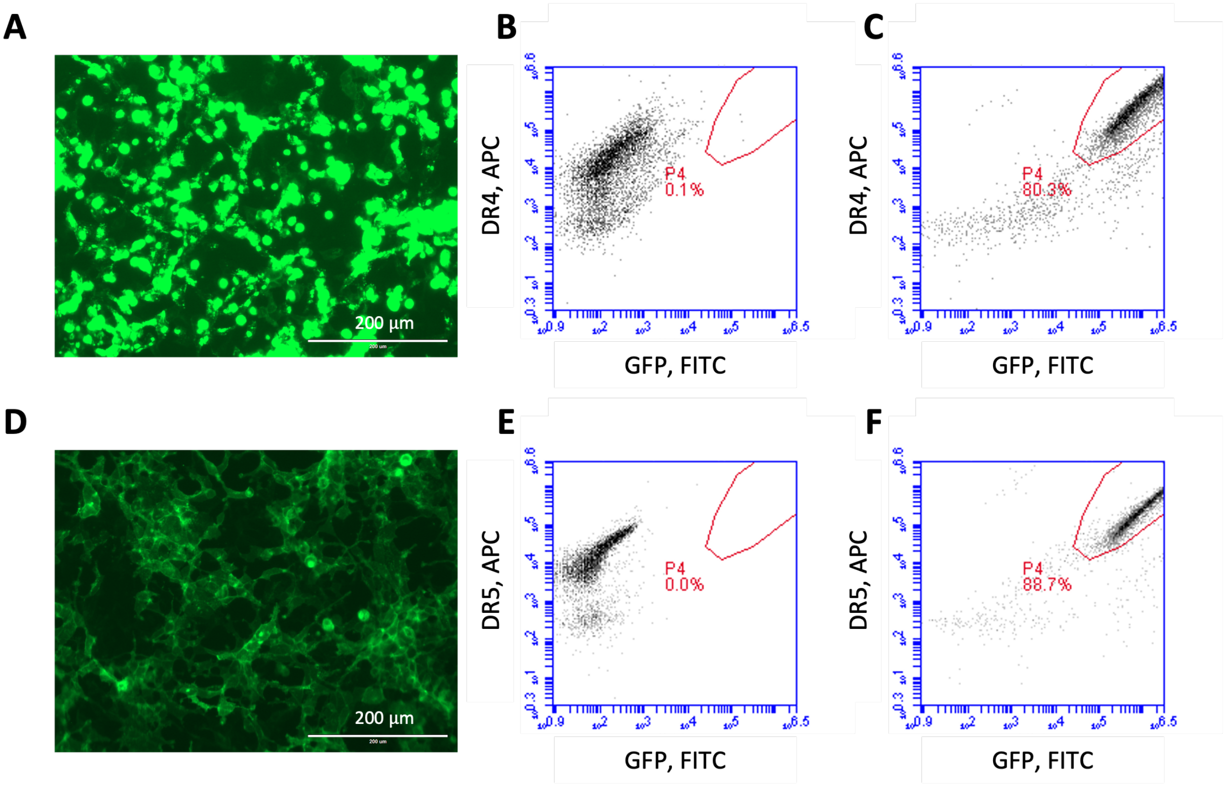

Figure S3. Antigens were successfully conjugated on the magnetic beads to perform MACS. (A) The expression signal of DR4-GFP in transiently transfected HEK293-DR4-GFP cells was observed under fluorescent microscopy. **(B)** GFP-trapping magnetic beads were labeled with 17 nM of goat-anti-mouse IgG pAb Alexa Fluor™ 647 conjugate. **(C)** GFP-trapping magnetic beads were incubated with lysate, and labeled with 17 nM of anti-hDR4 mAb and 17 nM of goat-anti-mouse IgG pAb Alexa Fluor™ 647 conjugate. **(D)** The expression signal of DR5-GFP in stably transfected HEK293-DR5-GFP cells was observed under fluorescent microscopy. **(E)** GFP-trapping magnetic beads were labeled with 17 nM of goat-anti-mouse IgG pAb Alexa Fluor™ 647 conjugate. **(F)** GFP-trapping magnetic beads were incubated with lysate, and labeled with 17 nM of anti-hDR5 mAb and 17 nM of goat-anti-mouse IgG pAb Alexa Fluor™ 647 conjugate.

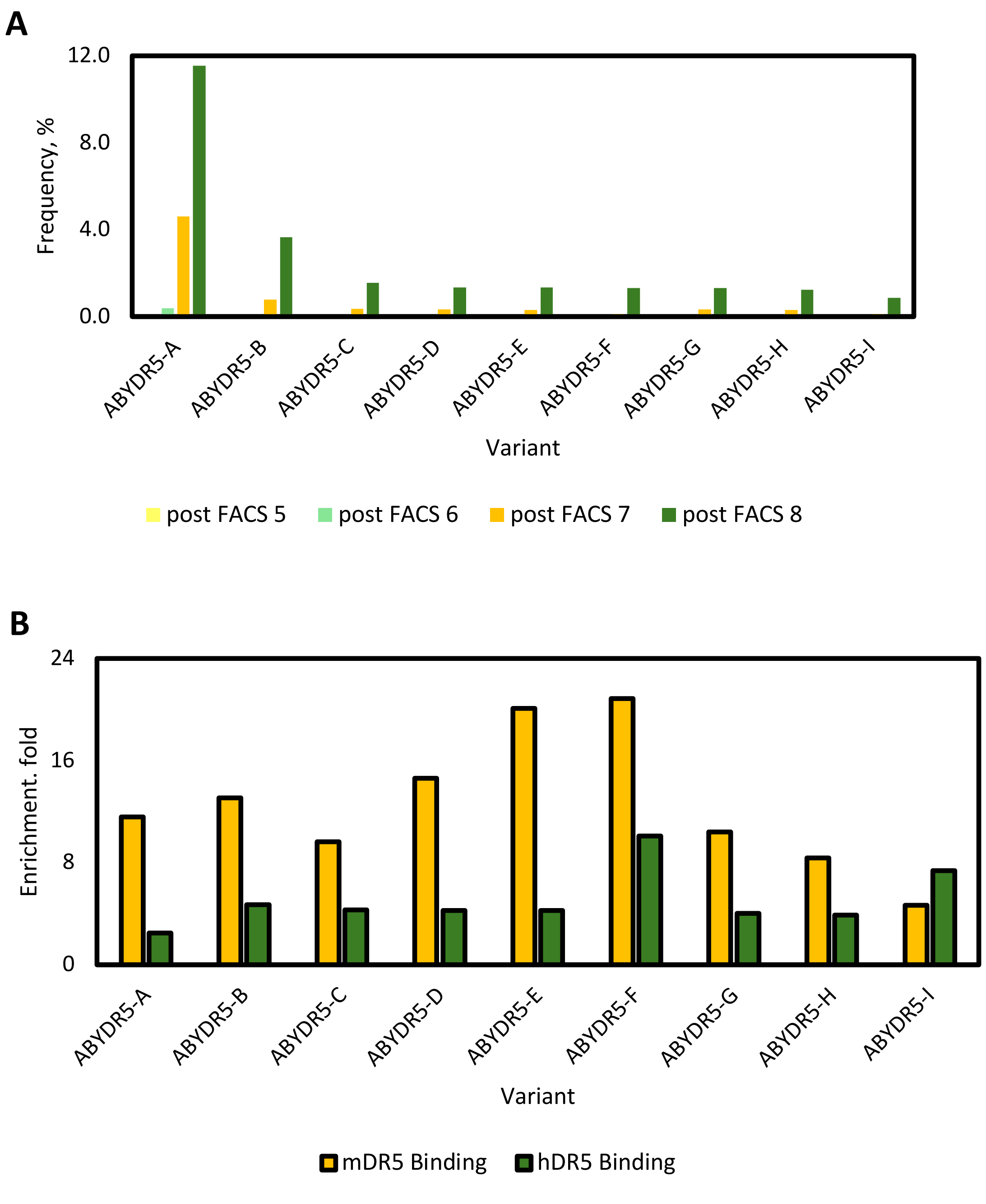

Figure S4. Enrichment of the lead affibodies in the final rounds of selection was quantified. (A) In 76,974 reads post FACS 5 mDR5, 31,945 reads post-FACS 6 hDR5, 35,955 reads post-FACS 7 mDR5, and 76,397 reads post FACS 8 mDR5, the frequency of the top 9 lead affibodies were assessed. Selections targeting murine DR5 were colored as yellow bars, and selections targeting human DR5 were shown as green bars. **(B)** Enrichment (frequency in FACS N ÷ FACS N-1) was calculated for the lead molecules in the final murine and human DR5 binding selections. Selections targeting murine DR5 were colored as yellow bars, and selections targeting human DR5 were shown as green bars.
